# Effect of sirolimus on muscle in inclusion body myositis observed with magnetic resonance imaging and spectroscopy

**DOI:** 10.1002/jcsm.13451

**Published:** 2024-04-13

**Authors:** Harmen Reyngoudt, Pierre‐Yves Baudin, Ericky Caldas de Almeida Araújo, Damien Bachasson, Jean‐Marc Boisserie, Kubéraka Mariampillai, Mélanie Annoussamy, Yves Allenbach, Jean‐Yves Hogrel, Pierre G. Carlier, Benjamin Marty, Olivier Benveniste

**Affiliations:** ^1^ NMR Laboratory, Neuromuscular Investigation Center Institute of Myology Paris France; ^2^ Neuromuscular Physiology and Evaluation Laboratory, Neuromuscular Investigation Center Institute of Myology Paris France; ^3^ INSERM, UMRS1158 Neurophysiologie Respiratoire Expérimentale et Clinique Sorbonne Université Paris France; ^4^ Department of Internal Medicine and Clinical Immunology, Inflammatory Myopathies Reference Center, Research Center in Myology UMR974, Sorbonne Université, Assistance Publique‐Hôpitaux de Paris Pitié‐Salpêtrière University Hospital Paris France; ^5^ I‐Motion Institute of Myology Paris France; ^6^ CEA, DRF, SHFJ University Paris‐Saclay Orsay France

**Keywords:** ^31^P MRS, Biomarkers, Inclusion body myositis, Quantitative MRI, Treatment

## Abstract

**Background:**

Finding sensitive clinical outcome measures has become crucial in natural history studies and therapeutic trials of neuromuscular disorders. Here, we focus on 1‐year longitudinal data from quantitative magnetic resonance imaging (MRI) and phosphorus magnetic resonance spectroscopy (^31^P MRS) in a placebo‐controlled study of sirolimus for inclusion body myositis (IBM), also examining their links to functional, strength, and clinical parameters in lower limb muscles.

**Methods:**

Quantitative MRI and ^31^P MRS data were collected at 3 T from a single site, involving 44 patients (22 on placebo, 22 on sirolimus) at baseline and year‐1, and 21 healthy controls. Assessments included fat fraction (FF), contractile cross‐sectional area (cCSA), and water T_2_ in global leg and thigh segments, muscle groups, individual muscles, as well as ^31^P MRS indices in *quadriceps* or *triceps surae*. Analyses covered patient‐control comparisons, annual change assessments via standard *t*‐tests and linear mixed models, calculation of standardized response means (SRM), and exploration of correlations between MRI, ^31^P MRS, functional, strength, and clinical parameters.

**Results:**

The *quadriceps* and *gastrocnemius medialis* muscles had the highest FF values, displaying notable heterogeneity and asymmetry, particularly in the *quadriceps*. In the placebo group, the median 1‐year FF increase in the quadriceps was 3.2% (*P* < 0.001), whereas in the sirolimus group, it was 0.7% (*P* = 0.033). Both groups experienced a significant decrease in cCSA in the *quadriceps* after 1 year (*P* < 0.001), with median changes of 12.6% for the placebo group and 5.5% for the sirolimus group. Differences in FF and cCSA changes between the two groups were significant (*P* < 0.001). SRM values for FF and cCSA were 1.3 and 1.4 in the placebo group and 0.5 and 0.8 in the sirolimus group, respectively. Water T_2_ values were highest in the *quadriceps* muscles of both groups, significantly exceeding control values in both groups (*P* < 0.001) and were higher in the placebo group than in the sirolimus group. After treatment, water T_2_ increased significantly only in the sirolimus group's *quadriceps* (*P* < 0.01). Multiple ^31^P MRS indices were abnormal in patients compared to controls and remained unchanged after treatment. Significant correlations were identified between baseline water T_2_ and FF at baseline and the change in FF (*P* < 0.001). Additionally, significant correlations were observed between FF, cCSA, water T_2_, and functional and strength outcome measures.

**Conclusions:**

This study has demonstrated that quantitative MRI/^31^P MRS can discern measurable differences between placebo and sirolimus‐treated IBM patients, offering promise for future therapeutic trials in idiopathic inflammatory myopathies such as IBM.

## Introduction

Inclusion body myositis (IBM) is the most common idiopathic inflammatory myopathy in individuals over 50 years old.^1^ While the exact cause remains uncertain, it is believed to result from a complex interplay of genetic, immune system, and environmental factors.^1^ IBM is distinguished by endomysial inflammation and highly differentiated cytotoxic T‐cells that destroy non‐necrotic myofibers, strengthening the hypothesis of an autoimmune origin to IBM.^2^, ^3^ A recent IBM autoimmune pathogenesis model suggests that chronic T‐cell activation leads to a series of events, including endoplasmic reticulum stress, mitochondrial and proteasome dysfunction, resulting in the abnormal accumulation of unfolded proteins and the formation of ‘inclusion bodies’, ultimately causing myofiber damage.^4^ Clinical diagnostic features include muscle involvement asymmetry and weakness in the *quadriceps* and finger *flexors*.^3^, ^5^

Currently, there is no cure for IBM, and treatment primarily focuses on symptom management and improving quality of life.^6^ Immunosuppressive therapies have shown limited effectiveness in IBM, possibly due to the unique nature of the T‐cell population involved.^2^, ^4^ Sirolimus, also known as rapamycin, is an mTOR inhibitor that targets the mammalian target of rapamycin (mTOR), a critical protein kinase regulating cell growth, proliferation, and survival.^7^ Sirolimus has found applications in various clinical and research contexts, such as immunosuppression to prevent kidney transplant rejection,^8^ cell growth inhibition in various cancer types,^9^ and influencing cellular senescence and inflammation in aging‐related research.^10^

In IBM studies, clinical outcome measures and biomarkers for evaluating disease status, disease progression and treatment efficacy have included a combination of functional and strength measures, questionnaires of function, mobility and quality of life, imaging and in some cases a biopsy.^4^, ^5^, ^11^ Magnetic resonance imaging (MRI) is the predominant imaging method used in these studies. Typically, MRI protocols consist of qualitative T_1_ and fat‐suppressed T_2_‐weighted MRI to evaluate muscle fat replacement, muscle atrophy, and changes in intramuscular water distribution due to active muscle damage, especially inflammation.^12^, ^13^, ^14^, ^15^, ^16^ More recent studies have incorporated quantitative MRI techniques, such as muscle fat fraction (FF) and T_2_ mapping,^17^ with some exploring longitudinal changes.^18^, ^19^, ^20^, ^21^, ^22^ Because repeated biopsies for monitoring disease progression are unrealistic, quantitative MRI and magnetic resonance spectroscopy (MRS) are now commonly used in many follow‐up studies of neuromuscular diseases to non‐invasively assess both acute and chronic pathological changes. The ultimate goal is to use these objective MRI/S‐based quantitative biomarkers as surrogate outcome measures in clinical trials.

In the study of Benveniste et al., evaluating sirolimus in IBM, the primary endpoint, a relative percentage change in maximal voluntary isometric knee extension strength at 12 months, showed no difference between placebo and sirolimus groups.^21^ However, secondary outcome measures in the same study revealed significant differences, including a smaller decline in 6‐min walking distance (6MWD) and a lesser increase in global thigh muscle fat fraction (FF) as measured by quantitative MRI in the sirolimus‐treated patients.

Our study examines the complete quantitative MRI and phosphorus MRS (^31^P MRS) data from the clinical phase‐2b trial of sirolimus as described by Benveniste et al.,^21^ and their relationship with muscle function and strength.

## Methods

### Subjects, study set‐up, and ethics

In a randomized, double‐blind, placebo‐controlled phase 2b trial (NCT02481453), data were collected from patients diagnosed with IBM based on established criteria.^4^ The complete study design, patient inclusion/exclusion criteria, randomization procedure, and adverse events are described in Benveniste et al.^21^ Control MRI and ^31^P MRS data were acquired from healthy individuals.

All participants provided written informed consent, adhering to the 1964 Declaration of Helsinki and its subsequent amendments. Healthy control subjects were scanned as part of an MRI/S protocol approved by the local ethics committee (Comité de Protection des Personnes [CPP] ‐ Ile de France VI ‐ Groupe Hospitalier Pitié‐Salpêtrière, ID RCB: 2012‐A01689‐34).

### Quantitative magnetic resonance imaging and phosphorus magnetic resonance spectroscopy data acquisition

Data were acquired using a 3‐T MRI clinical scanner (Prisma^Fit^, Siemens Healthineers, Erlangen, Germany). For quantitative MRI, the local system's body radiofrequency (RF) coil was used for signal transmission and surface‐array RF coils for signal reception. Patients were positioned supine with feet first, and MRI sequences were centred at one‐third of the femur (distally) and at the widest part of the calf, scanning both sides. For ^31^P MRS, a flexible transmit/receive ^1^H/^31^P surface RF coil (11 cm, Rapid Biomedical GmbH, Rimpar, Germany) was used. A standardized procedure ensured consistent subject and RF coil placement, maintaining identical acquisition parameters throughout the study.

Quantitative water‐fat imaging was performed using a 3D gradient echo (GRE) sequence with three‐point Dixon technique: echo times (TE) = 2.75 − 3.95 − 5.15 ms, repetition time (TR) = 10 ms, 1 × 1 mm^2^ in‐plane resolution, 64 slices of 5 mm covering 320 mm for both thighs and legs. For T_2_ mapping, a 2D multi‐spin‐echo (MSE) sequence was utilized: 17 equidistant TEs (9.5–161.5 ms), TR = 3000 ms, 1.4 × 1.4 mm^2^ resolution, 9 slices of 10 mm, 30 mm slice gap (covering the 3D volume from the three‐point‐Dixon sequence).^23^ A B_1_ map sequence was acquired to determine the transmit field (B_1_^+^) in each voxel. ^31^P MRS data were obtained in the anterior part of the right thigh (*quadriceps*) or the posterior part of the right leg (*triceps surae*) using a non‐localized free‐induction decay of 64 averages (TR of 4000 ms, bandwidth of 3000 Hz, 2048 data points).^24^ The ^1^H/^31^P surface RF coil was placed around the *quadriceps* if muscle fat replacement was <30% (i.e., Lamminen‐Mercuri/LM grade 1/2^25^) or around the calf covering the *triceps surae* (if muscle fat replacement was >30% or LM grade 3 in *quadriceps*). Total acquisition time for quantitative MRI and ^31^P MRS was approximately 50 min.

### Quantitative magnetic resonance imaging and phosphorus magnetic resonance spectroscopy data processing

An experienced MRI technician manually defined regions of interest (ROIs) using the itksnap (http://www.itksnap.org) software tool in seven leg muscles (*extensor digitorum longus*, *tibialis anterior*, *tibialis posterior*, *peroneus longus*, *soleus*, and *gastrocnemius medialis/lateralis*) and 11 thigh muscles (*vastus lateralis/intermedius/medialis*, *rectus femoris*, *gracilis*, *sartorius*, *adductor magnus*/*longus*, long head of *biceps femoris*, *semimembranosus*, and *semitendinosus*) on the first TE‐image of the central five slices of the MSE series. The ROIs accurately outlined the interior of the muscle, avoiding visible fasciae and major blood vessels. Additionally, muscle group ROIs (thigh: *quadriceps*, hamstring; leg: anterior leg or *extensor*, *triceps surae*) and global muscle segment ROIs (thigh and leg), accurately following muscle boundaries, were defined on the out‐of‐phase Dixon images (same five slices).^26^

FF maps were reconstructed from GRE images using the three‐point Dixon method. FF, calculated as SI (fat)/((SI)fat + (SI)water)·100, where SI represents signal intensity, was determined for all ROIs, excluding FF maps with partial fat‐water swaps. Cross‐sectional area (CSA) values were computed for muscle group and global segment ROIs. Contractile CSA (cCSA), representing the part of the ROI containing the contractile apparatus, was calculated using the equation cCSA = CSA·(1 − (0.01·FF)). To account for body size, allometric scaling of cCSA was performed using body surface area, taking into account individual height and weight (see supporting information for calculation^27^). Disease progression was assessed by baseline‐year‐1 changes in FF (ΔFF, %) and cCSA (ΔcCSA_rel_, relative difference, %).^28^

Water T_2_ maps were reconstructed from the MSE data using a tri‐exponential fitting procedure, considering water and lipids signals.^23^ Only pixels with B_1_^+^ values between 80% and 120% of the prescribed flip angle were analysed. ROIs with fewer than 10 pixels were excluded from analysis. Water T_2_ values were determined in individual muscle ROIs. Weighted water T_2_ values were calculated for muscle groups and global segments based on individual muscle sizes. Baseline‐year‐1 changes in water T_2_ (ΔT_2_, ms) were also assessed.

All quantitative MRI data were processed using in‐house developed Python code.

^31^P MRS data were processed as described previously^24^ using jMRUI^29^ and Topspin (Bruker Medical GMbG, Ettlingen, Germany) to calculate various ratios, including P_i,tot_/PCr (total inorganic phosphate over phosphocreatine), P_i,b_/P_i,tot_ (alkaline Pi over P_i,tot_), P_i,tot_/γATP (P_i,tot_ over adenosine triphosphate), PCr/γATP, PDE/γATP (phosphodiesters over γATP), and PME/γATP (phosphomonoesters over γATP), as well as values for pH_w_ (weighted pH, based on the relative weights of cytosolic P_i_ and alkaline P_i_ resonances) and intramuscular magnesium concentration [Mg^2+^]. Only ^31^P MRS data with sufficient signal‐to‐noise ratio (SNR > 10 for PCr resonance) were included for analysis.

### Function, strength, and creatine kinase measurements

For a comprehensive description of the various functional and strength assessments and results, we refer to Benveniste et al.^21^ In this study, we explored the potential relationships between MRI/^31^P MRS‐based quantitative biomarkers and the following clinical outcome measures: 6MWD (in meters, m, or percentage of predicted normal values for age, %pred^5^), maximal voluntary isometric knee extension strength (in Newton meters, Nm, or %pred), knee flexion strength (in Nm, or %pred), 1‐year changes (∆6MWD, ∆knee extension, ∆knee flexion),^4^ and creatine kinase (CK) concentration (in units per litre, U/L).

### Statistical analysis

SPSS software Version 22 (SPSS, Chicago, IL, USA) was used for statistical analyses. Mann–Whitney tests were used for comparisons between patients and controls, and between patient groups. Wilcoxon tests were employed for right–left and baseline‐year‐1 comparisons.

A linear mixed model, with segment, muscle group, or muscle as a within‐subject factor, was utilized to compare ΔFF, ΔcCSA_rel_, and ΔT_2_ between the placebo and sirolimus groups.

The Spearman rank correlation test explored relationships between MRI/^31^P MRS variables and clinical, functional, and strength measures.

A significance level of *P* < 0.05 was corrected for multiple (surrogate) outcome measures (MRI: *P* = 0.05/3 = 0.017; ^31^P MRS: *P* = 0.05/8 = 0.006).

To assess sensitivity to change over time, standardized response means (SRM) were calculated for FF and cCSA (SRM ≥ 0.8 indicates high sensitivity to change, based on Cohen thresholds). For water T_2_ and ^31^P MRS indices, both SRM and standardized difference means (SDM) were evaluated.^28^

## Results

### Data overview and demographics

**Figure 1:**
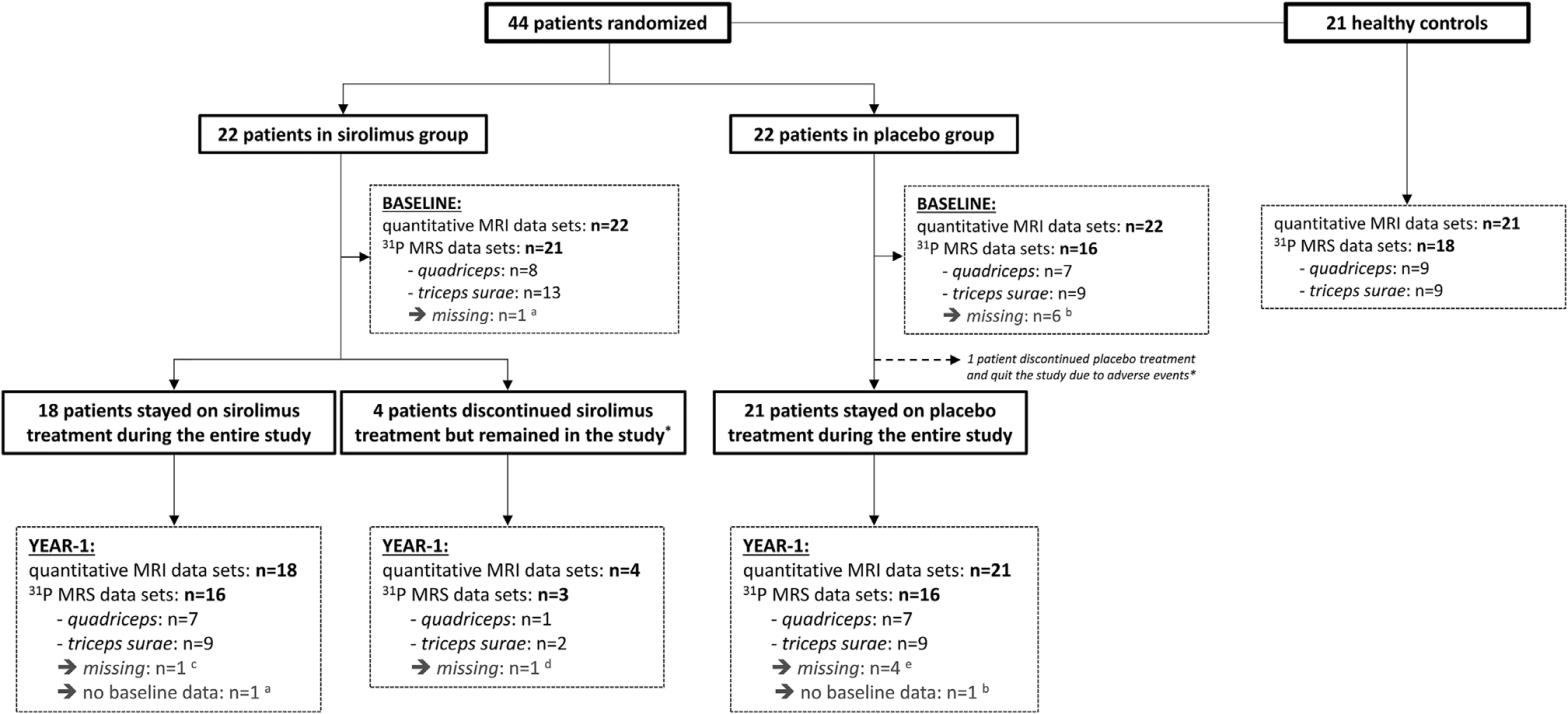
Flow chart of the acquired quantitative MRI and ^31^P MRS data in the current study. All but one patient underwent both baseline and year‐1 visits, with one patient (from the placebo group) quitting the study prematurely following adverse events. From the 22 patients in the sirolimus group, 18 subjects stayed on sirolimus for the entire study and 4 subjects stopped sirolimus treatment but remained in the study. From the 44 baseline and 43 year‐1 quantitative MRI data sets, one Dixon series in leg was excluded from further analysis due to partial fat‐water swaps. All 37 baseline and 35 year‐1 ^31^P MRS data sets were of sufficient quality for further analysis. *For details on adverse events, we refer to the work of Benveniste et al.^21^ ^a^No baseline ^31^P MRS data due to a technical problem with RF antenna (patient 44). ^b^No baseline ^31^P MRS data due to a technical problem with RF antenna (patient 27) and too high FF in both *quadriceps* and *triceps surae* (patients 3, 5, 13, 16 and 20). ^c^No year‐1 ^31^P MRS data due to a technical problem with RF antenna (patient 1). ^d^No year‐1 ^31^P MRS data due to medical reasons (patient 22). ^e^No year‐1 ^31^P MRS data due to too high FF in both quadriceps and *triceps surae* (patients 3, 13, 16 and 20).

Figure 1 provides an overview of the acquired data sets. Table 1 outlines key demographic, clinical, and functional distinctions between healthy controls and patients at baseline. Healthy controls were slightly younger but were not significantly different for sex or BMI compared with patients. At baseline, no significant differences were observed between the placebo and sirolimus patient groups. For individual patient data, we refer to Table S1.

**Table 1:** Demographic, clinical, and functional data in controls and patients

	Controls	Patients placebo (at baseline)	Patients sirolimus (at baseline)	*P* ^a^	*P* ^b^
*n*	21	22	22		
Female/male	11/10	12/10	8/14	0.481	0.296
Age (years)	59.0 [54.1–61.1]	66.7 [58.4–73.8]	68.1 [62.8–73.9]	**0.009** **	0.716
BMI (kg/m^2^)	24.6 [21.1–26.3]	24.2 [22.6–26.8]	24.4 [22.8–26.6]	0.762	0.865
Years since symptom onset (years)	n/a	7.4 [3.8–10.7]	7.0 [4.2–10.5]	‐	0.981
Years since diagnosis (years)	n/a	2.8 [1.1–5.6]	1.9 [0.5–4.4]	‐	0.343
CK (U/L)	n/d	446.0 [276.3–777.3]	314.5 [217.5–652.5]	‐	0.552
6MWD (m)	n/d	324.0 [264.5–417.3]	392.5 [354.0–552.5]	‐	0.063
Knee flexion strength (Nm)	n/d	28.8 [19.8–43.1]	33.7 [23.2–46.6]	‐	0.546
Knee extension strength (Nm)	n/d	22.9 [10.8–46.1]	22.1 [15.1–41.9]	‐	0.782

BMI, body mass index (kg/m^2^); CK, creatine kinease concentration (U/L); n, number of subjects; n/a, not applicable; n/d, not determined; 6MWD, 6‐min walking distance (in m).

* *P* < 0.017.

** *P* < 0.01.

*** *P* < 0.001 (significant differences with controls and between baseline‐year 1).

^a^ Difference between controls and patient.

^b^ Difference between placebo and sirolimus groups at baseline. Results are indicated with a median value and the inter‐quartile distance between square brackets.

### Differences with controls

**Figure 2:**
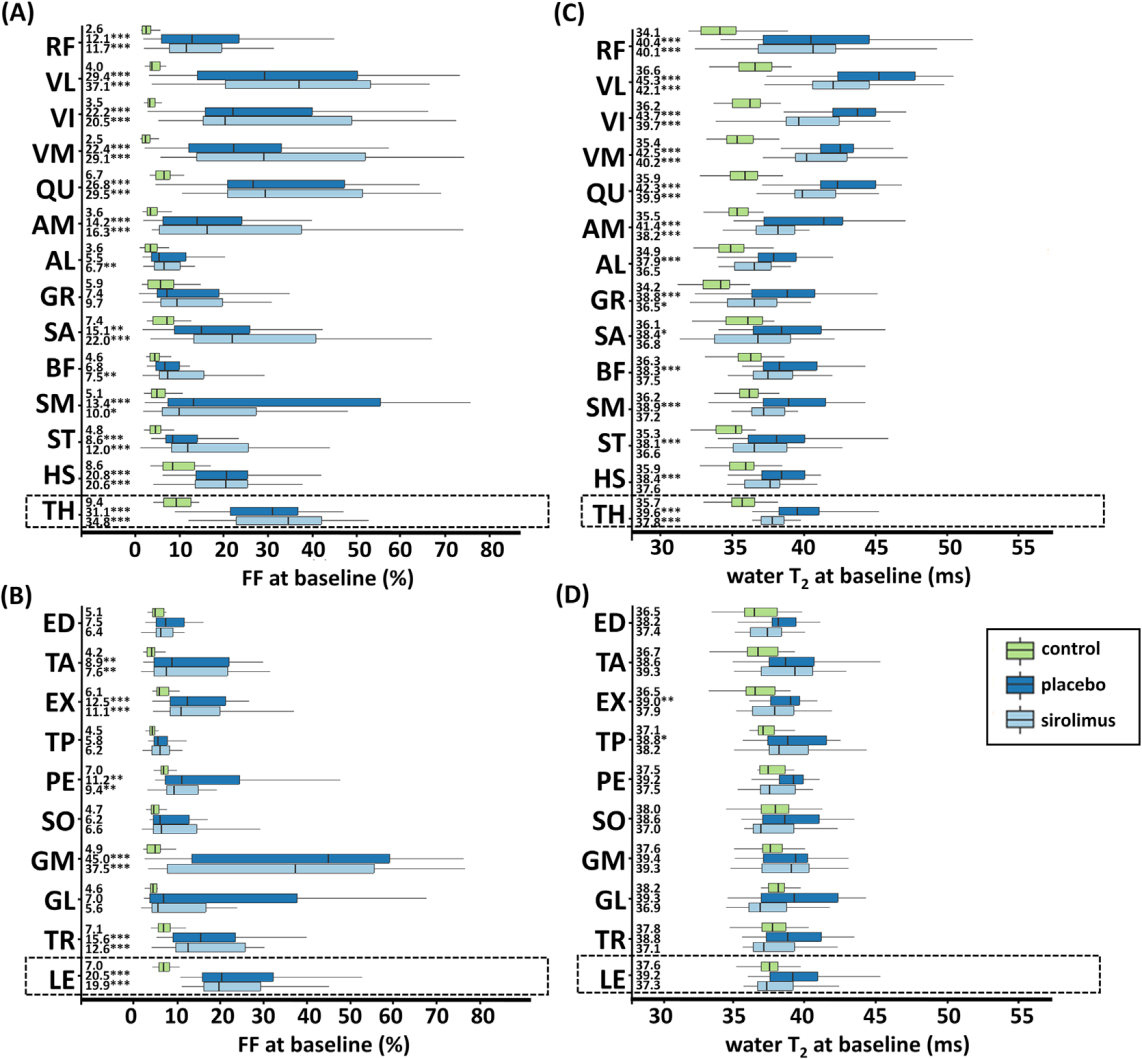
Box‐and‐whisker plots of baseline quantitative MRI data in all regions of interest (ROI) in controls and patients. (A) FF in thigh. (B) FF in leg. (C) Water T_2_ in thigh. (D) Water T_2_ in leg. Median FF values are indicated per ROI per subject group. *P < 0.017; **P < 0.01; ***P < 0.001 (significant differences between patients and controls). AL, *adductor longus*; AM, *adductor magnus*; BF, *biceps femoris* (long head); ED, *extensor digitorum longus*; EX, *extensor*; FF, fat fraction (%); GL, *gastrocnemius lateralis*; GM, *gastrocnemius medialis*; GR, *gracilis*; HS, hamstring; LE, global leg; PE, *peroneus longus*; QU, *quadriceps*; SA, *sartorius*; SM, *semimembranosus*; SO, *soleus*; ST, *semitendinosus*; TA, *tibialis anterior*; TH, global thigh; TR, *triceps surae*; TP, *tibialis posterior*; VI, *vastus intermedius*; VM, *vastus medialis*; VL, *vastus lateralis*.

FF values significantly differed from controls in nearly all muscles, muscle groups, and global segments (Figure 2A,B, Table S2). Values for cCSA were also significantly different in all muscle groups and global segments (Figure S1A, Table S3). Water T_2_ values showed significant differences with controls only in the thigh (Figure 2C,D, Table S4). Several ^31^P MRS indices were notably abnormal in patients versus controls, including elevated P_i,tot_/PCr and PDE/γATP in *triceps surae* (*P* < 0.001), increased PME/γATP in *triceps surae* and *quadriceps* (*P* < 0.001), and increased pH_w_ in *quadriceps* (*P* = 0.002). Further details are available in Table S5.

### Baseline quantitative magnetic resonance imaging

In patients, significant left–right differences in FF were found in *quadriceps* (e.g., *vastus lateralis*: *P* < 0.001; *quadriceps*: *P* < 0.001), hamstring (e.g., *semimembranosus*: *P* < 0.001), and *triceps surae* (e.g., *soleus*: *P* < 0.001; *triceps surae*: *P* = 0.013). Asymmetries for water T_2_ were mainly observed in medio‐posterior thigh muscles (e.g., *semimembranosus*: *P* < 0.001), with no left–right differences for cCSA. See Table S6 for detailed results. Nevertheless, all subsequent analyses were based on mean values for left and right.

There were no significant differences between the placebo and sirolimus patient groups for FF (*P* > 0.126 for all, Figure 2A,B) and cCSA (*P* > 0.290 for all, Figure S1A). However, median FF values were, in general, slightly higher in the sirolimus group, especially in *quadriceps* muscles. Median water T_2_ values were higher in the placebo group, though the differences with the sirolimus group were not significant (*P* > 0.130 for all, Figure 2C,D). Detailed baseline FF, cCSA, and water T_2_ results for patients can be found in Tables S2, S3, and S4, respectively.

**Figure 3:**
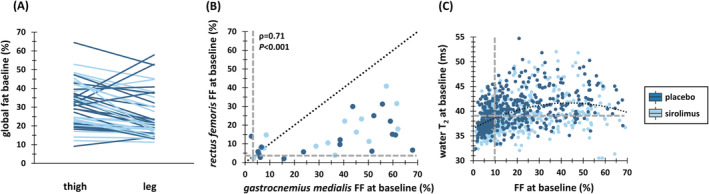
Baseline quantitative MRI features in IBM. (A) Global thigh and leg FF profile per individual patient: In only five patients there was a stronger distal involvement (i.e., higher global leg FF than global thigh FF). (B) Relationship between baseline FF in *gastrocnemius medialis* and baseline FF in *rectus femoris* for all patients (dotted line = identity line; dashed lines depict the 90^th^ percentile values in controls for baseline FF: 2.8% for *gastrocnemius medialis* and 4.2% for *rectus femoris*). (C) Relationship between baseline water T_2_ and baseline FF for all regions of interest and all patients (dashed lines depict the 90^th^ percentile values in controls: 9.8% for FF and 38.7 ms for water T_2_). Data were better fitted by 2^nd^ degree polynomial function (T_2_ = 36.5 + 0.24·FF −0.003·FF^2^, R^2^ = 0.14, *P* < 0.001) than by a linear function (T_2_ = 37.7 + 0.08·FF, R^2^ = 0.19, *P* < 0.001). FF, fat fraction (%); R^2^, correlation coefficient.

When examining the baseline quantitative MRI data, *gastrocnemius medialis* and *quadriceps* muscles, except for the relatively spared *rectus femoris*, showed the most significant disease involvement based on FF and cCSA. When global thigh FF was below 20%, thigh and leg exhibited similar global FF values. However, in most cases, global thigh FF was higher than global leg FF, and only five patients showed a stronger leg involvement (Figure 3A). A strong correlation was observed between *gastrocnemius medialis* and *rectus femoris*, two muscles of interest in IBM disease progression (Figure 3B). *Quadriceps* muscles had particularly elevated water T_2_ values. There was a significant positive relationship between FF and water T_2_ (Figure 3C, Table S7).

### Longitudinal quantitative magnetic resonance imaging

**Figure 4:**
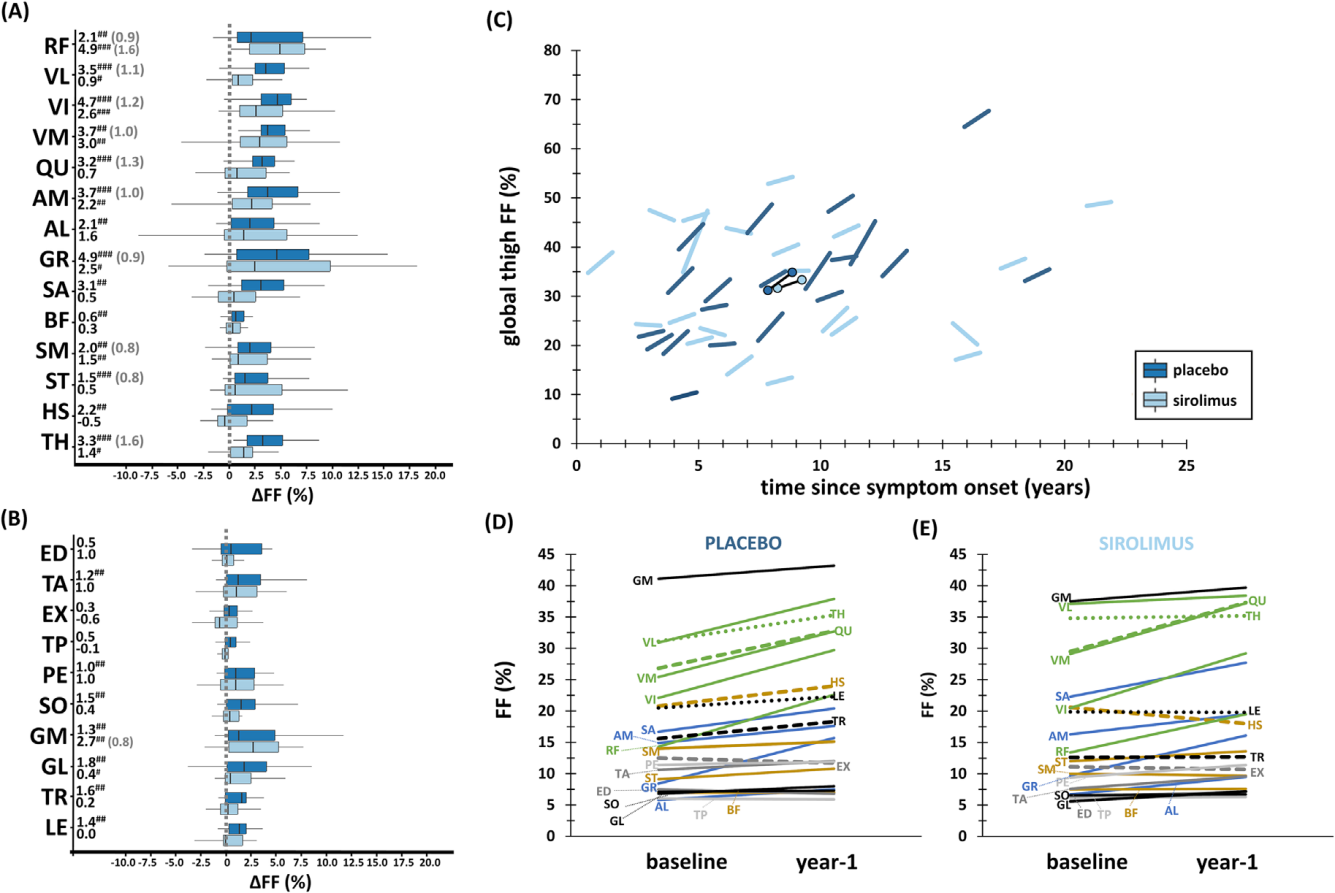
Year‐1 changes in FF. (A) Box‐and‐whisker plots of ∆FF in thigh regions of interest (median values and SRM values ≥0.8 per group are indicated on the left). (B) Box‐and‐whisker plots of ∆FF in leg regions of interest (median values and SRM values ≥0.8 per group are indicated on the left). (C) Global thigh FF trajectories for individual patients as a function of time since symptom onset (median values per group are also depicted, black lines). (D) Median FF per region of interest at baseline and year‐1 in placebo group. (E) Median FF per region of interest at baseline and year‐1 in sirolimus group. ^#^ *P* < 0.017; ^##^ *P* < 0.01; ^###^ *P* < 0.001 (significant differences between baseline and year‐1). AL, *adductor longus*; AM, *adductor magnus*; BF, *biceps femoris* (long head); ED, *extensor digitorum longus*; EX, *extensor*; FF, fat fraction (%); GL, *gastrocnemius lateralis*; GM, *gastrocnemius medialis*; GR, *gracilis*; HS, hamstring; LE, global leg; PE, *peroneus longus*; QU, *quadriceps*; SA, *sartorius*; SM, *semimembranosus*; SO, *soleus*; SRM, standardized response mean; ST, *semitendinosus*; TA, *tibialis anterior*; TH, global thigh; TR, *triceps surae*; TP, *tibialis posterior*; VI, *vastus intermedius*; VM, *vastus medialis*; VL, *vastus lateralis*; ∆FF, 1‐year change in FF (%).

Figure S2 depicts examples for baseline and year‐1 quantitative FF maps from one placebo‐ and one sirolimus‐treated patient with similar baseline FF. The increase in FF was generally more pronounced in the placebo group (significant for nearly all ROIs) compared with the sirolimus group (Figure 4A,B, Table S2). Significant differences in ∆FF were observed for global thigh segments (placebo: ∆FF = 3.3%; sirolimus: ∆FF = 2.0%; *P* = 0.008), thigh muscle groups (*P* < 0.001), and individual thigh muscles (*P* = 0.003). This faster disease progression in the placebo group was also evident from the individual patient trajectories for global thigh FF (Figure 4C). The greatest ∆FF values in individual muscles were found in the placebo group for *quadriceps muscles*, especially in *vastus lateralis* (Figures 4D,E and S3). Only for *rectus femoris* and *gastrocnemius medialis*, a higher ∆FF was observed in the sirolimus group. SRM values in the thigh were systematically higher in the placebo group and were nearly all lower than 0.8 for the sirolimus group.

Both placebo and sirolimus groups experienced a significant decrease in *quadriceps*, hamstring, and global thigh cCSA (Figure S1B, Table S3). Significant differences in ∆cCSA_rel_ between groups were observed for some muscle groups, especially for *quadriceps* (placebo: ∆cCSA_rel_ = −10.9%; sirolimus: ∆cCSA_rel_ = −6.9%; *P* < 0.001). SRM values equal to or higher than 0.8 for cCSA were only found in the thigh in the placebo group.

**Figure 5:**
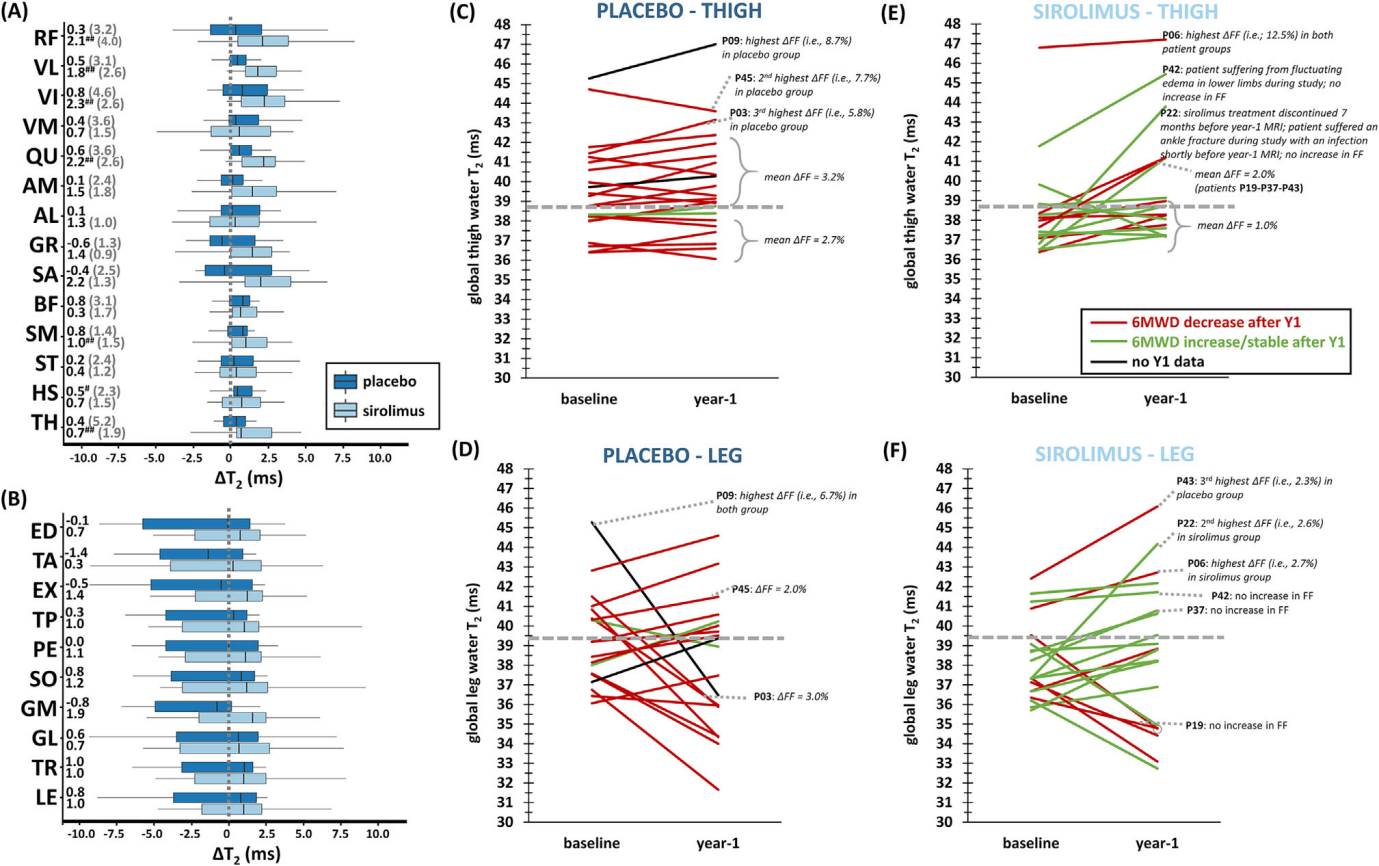
Year‐1 changes in water T_2_. (A) Box‐and‐whisker plots of ∆T_2_ in thigh regions of interest (median values and SDM values ≥0.8 per group are indicated on the left). (B) Box‐and‐whisker plots of ∆T_2_ in leg regions of interest (median values per group are indicated on the left). (C) Global thigh water T_2_ at baseline and year‐1 for individual patients in placebo group. (D) Global leg water T_2_ at baseline and year‐1 for individual patients in placebo group. (E) Global thigh water T_2_ at baseline and year‐1 for individual patients in sirolimus group. (F) Global leg water T_2_ at baseline and year‐1 for individual patients in sirolimus group. The horizontal dashed lines in plots C, D, E and F depict the 90^th^ percentile as determined in controls (i.e., 38.7 ms for thigh, 39.4 ms for leg). Several individual results are further explained in the plots, for patients P03, P09 and P45 for the placebo group and for patients P06, P22 and P42 for the sirolimus group. Patient P19 had four falls during the study with a right ankle sprain 2 weeks before the year‐1 MRI. Patient P37 suffered from persistent oedema in the lower limbs and a left ankle sprain following a fall during the study. Patient P43 also suffered from persistent oedema in the lower limbs in the 6 months before the year‐1 MRI and also suffered from a fracture 3 months before the year‐1 MRI. For thighs, mean ∆FF values are shown for the patients with water T_2_ < 38.7 ms (both groups), patients with water T_2_ > 38.7 ms (placebo group) and for the individual patients mentioned earlier. ^#^ *P* < 0.017; ^##^ *P* < 0.01; ^###^ *P* < 0.001 (significant differences between baseline and year‐1). AL, *adductor longus*; AM, *adductor magnus*; BF, *biceps femoris* (long head); ED, *extensor digitorum longus*; EX, *extensor*; GL, *gastrocnemius lateralis*; GM, *gastrocnemius medialis*; GR, *gracilis*; HS, hamstring; LE, global leg; PE, *peroneus longus*; QU, *quadriceps*; SA, *sartorius*; SDM, standardized difference mean; SM, *semimembranosus*; SO, *soleus*; ST, *semitendinosus*; TA, *tibialis anterior*; TH, global thigh; TR, *triceps surae*; TP, *tibialis posterior*; VI, *vastus intermedius*; VM, *vastus medialis*; VL, *vastus lateralis*; ∆FF, 1‐year change in FF (%); ∆T_2_, 1‐year change in water T_2_ (ms).

In the sirolimus group, water T_2_ values increased significantly compared with baseline in *quadriceps* (*P* < 0.01), while water T_2_ remained stable in the placebo group (Figure 5A,B, Table S4). Significant differences in ∆T_2_ between groups were noted for individual muscles (*vastus lateralis* placebo: ∆T_2_ = 0.7 ms; *vastus lateralis* sirolimus: ∆T_2_ = 2.0 ms; *P* < 0.001), global segments (*P* = 0.010), and muscle groups (*P* = 0.002). Further analysis of water T_2_ changes on a per‐patient basis revealed that the significant increase in water T_2_ in the sirolimus group was primarily due to a small group of patients (Figure 5C–F). Both in placebo and sirolimus groups, there was a group of patients where water T_2_ decreased in leg muscles after 1 year, but this was not significant overall (Figure 5D–F). Overall, SRM values for water T_2_ were low, but SDM values were high in the thigh (≥0.8). Figure 5C–F also illustrates that 6MWD, after 1 year, decreased in almost all placebo patients, and was stable or increased in the majority of the sirolimus patients.

### Phosphorus magnetic resonance spectroscopy

No significant baseline differences in ^31^P MRS indices were observed between the placebo and sirolimus groups (all *P* > 0.108). Additionally, there were no significant changes in these indices from baseline to year‐1 in both groups (in *triceps surae* and *quadriceps*, all *P* > 0.115). However, year‐1 values were significantly higher for P_i,tot_/PCr (*P* = 0.003) and P_i,tot_/γATP (*P* = 0.005) in *triceps surae* of the placebo group compared with the sirolimus group. Table S6 provides a comprehensive summary of all ^31^P MRS data.

### Early indicators of disease progression

**Figure 6:**
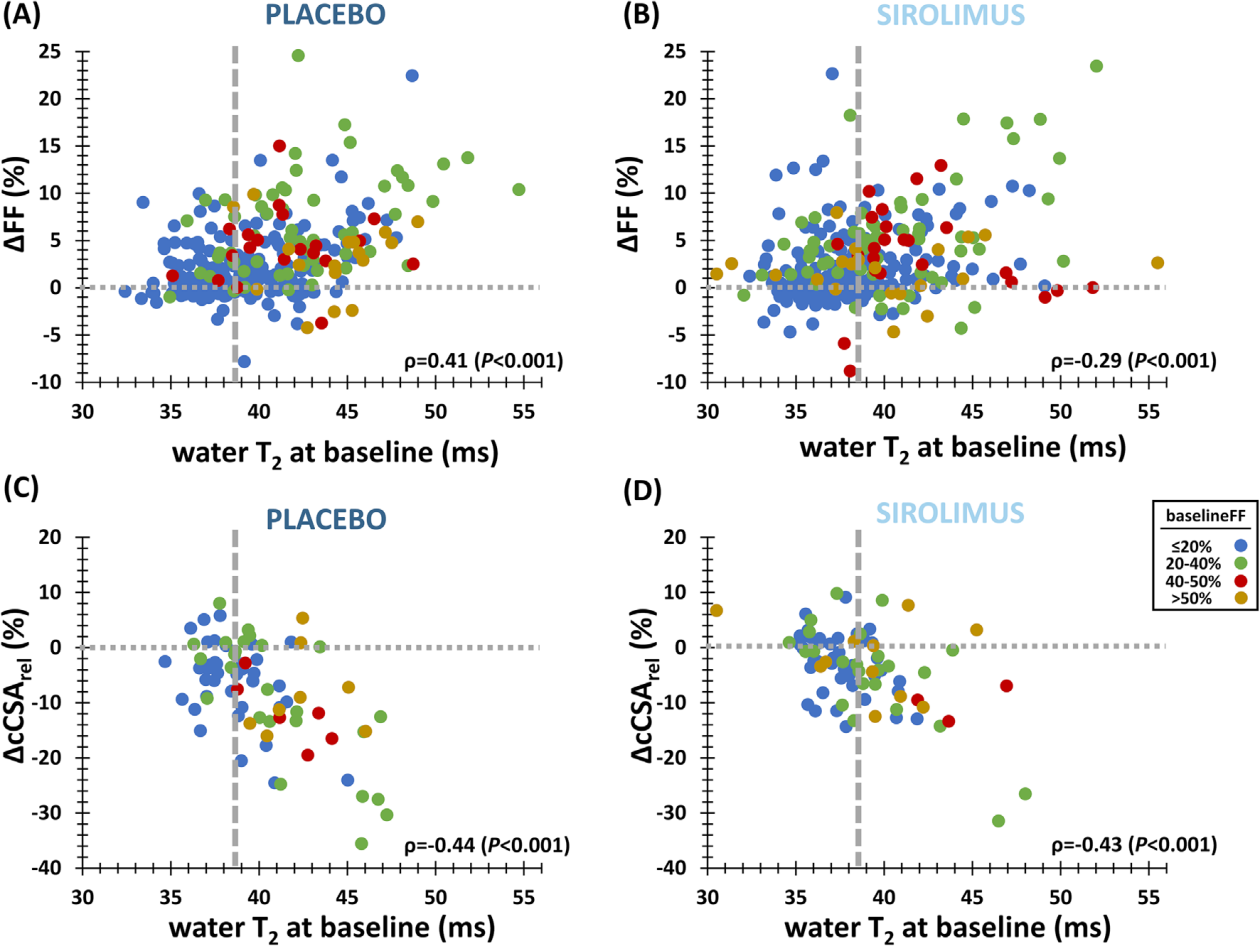
Correlations between water T_2_ and changes in FF and cCSA. (A) Relationship between baseline water T_2_ and changes in FF in all individual thigh and leg muscles in placebo group. (B) Relationship between baseline water T_2_ and changes in FF in all individual thigh and leg muscles in sirolimus group. (C) Relationship between baseline water T_2_ and changes in cCSA in thigh and leg muscle groups in placebo group. (D) Relationship between baseline water T_2_ and changes in cCSA in thigh and leg muscle groups in sirolimus group. Colours depict four different FF categories: Low (≤20%), moderate (20–40%), approximately half of muscle tissue replaced by fat (40–50%) and high (>50%). The vertical dashed lines depict the 90^th^ percentile value for water T_2_ across all thigh and leg muscles (i.e., 38.6 ms), as determined in controls. ∆cCSA_rel_, relative 1‐year change in cCSA (%); ∆FF, 1‐year change in FF (%).

A global analysis of all individual thigh and leg muscles revealed significant correlations between baseline water T_2_ and ΔFF in both placebo (Figure 6A) and sirolimus groups (Figure 6B). However, when investigating on a per‐muscle basis, this correlation was not significant for most muscles (especially for leg). Hamstring muscles were an exception, with the strongest correlation in *semitendinosus* (ρ = 0.70 and ρ = 0.68 in placebo and sirolimus groups, respectively, *P* < 0.001).

Similarly, a significant correlation was observed between baseline water T_2_ and ΔcCSA_rel_ in thigh and leg muscle groups in both placebo (Figure 6C) and sirolimus groups (Figure 6D). A trend, yet insignificant, was seen between baseline PME/γATP and ΔFF in *quadriceps*. Tables S7 and S8 provide a summary of correlations between various quantitative MRI and ^31^P MRS indices.

### Relationships with functional, strength and clinical parameters

The high SRM value for ∆FF and ∆cCSA_rel_ (in thigh muscles and thigh muscle groups) as found in the placebo group was also found for ∆6MWD (Table S9).

**Figure 7:**
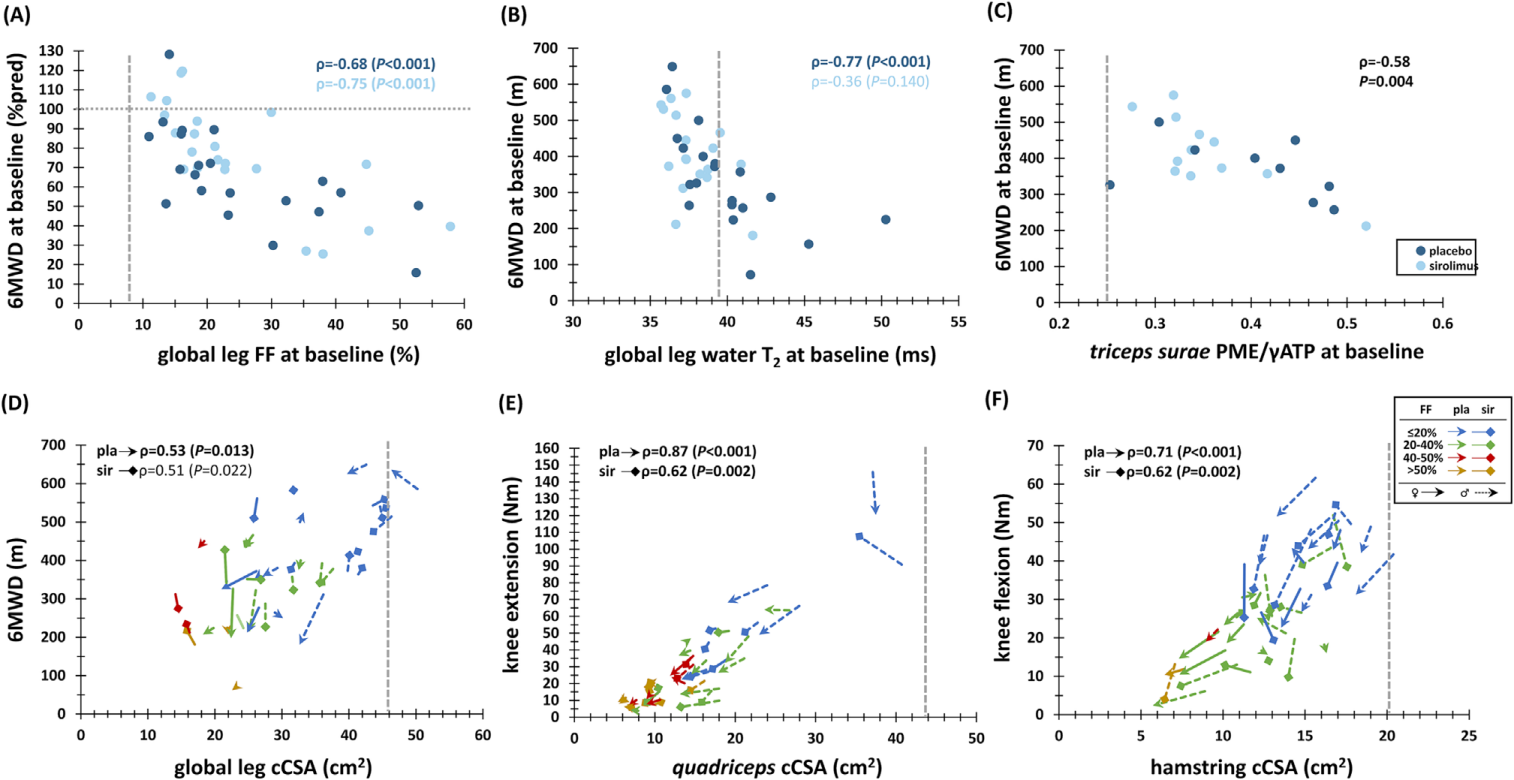
Correlations between quantitative MRI or ^31^P MRS biomarkers and functional/strength outcome measures. (A) Relationship between global leg FF and 6MWD (in %pred) at baseline. (B) Relationship between global leg water T_2_ and 6MWD (in m) at baseline. (C) Relationship between triceps surae PME/γATP and and 6MWD (in m) at baseline. (D) Relationship between global leg cCSA and 6MWD (in m). (E) Relationship between quadriceps cCSA and absolute knee extension strength (in Nm). (F) Relationship between hamstring cCSA and absolute knee flexion strength (in Nm). The vertical dashed lines depict the 90^th^ percentile value for global leg FF (i.e., 9.8%), global leg water T_2_ (i.e., 39.4 ms), *triceps surae* PME/γATP (i.e., 0.25), global leg cCSA (i.e., 45.9 cm^2^), quadriceps cCSA (i.e., 43.9 cm^2^), hamstring cCSA (i.e., 20.4 cm^2^), as determined in controls. Spearman rho (ρ) correlation values and corresponding *P*‐values are also indicated for both groups. cCSA, contractile cross‐sectional area (cm^2^); FF, fat fraction (%); PME, phosphomonoesters; ATP; adenosine triphosphate (γ refers to γ‐resonance of ATP in ^31^P MR spectrum); 6MWD, 6‐min walking distance (%pred or m).

At baseline, significant correlations were found between FF and 6MWD, spanning leg and thigh global segments, muscle groups, and most individual leg and thigh muscles (ρ: −0.45 to −0.75), with the strongest association in the global leg segment in both placebo and sirolimus groups (Figure 7A). In leg muscles, leg muscle groups, and the global leg of the placebo group only, water T_2_ exhibited a significant correlation with 6MWD (ρ: −0.46 to −0.77, Figure 7B), but not in the thigh. In *triceps surae*, but not in *quadriceps*, baseline PME/γATP was significantly correlated with 6MWD when combining both groups (Figure 7C).

For cCSA and 6MWD, significant correlations were only found in global leg (Figure 7D) and hamstring (ρ = 0.53), in the placebo group only. Strong to very strong correlations were found between knee extension strength and various quantitative MRI parameters, particularly for *quadriceps* FF (ρ = −0.74/−0.64 for placebo/sirolimus) and cCSA (Figure 7E). Similarly, significant strong correlations were observed between knee flexion strength and quantitative MRI parameters in hamstring for cCSA (Figure 7F) and FF (ρ = −0.78/−0.74 for placebo/sirolimus).

A significant correlation between ΔFF and Δ6MWD was found for global leg (Figure S4A) and global thigh (ρ = −0.66, *P* = 0.003), only in the placebo group. In several thigh muscles and thigh muscle groups, baseline water T_2_ showed significant correlations with Δ6MWD, with the most pronounced correlation found in the *vastus lateralis* muscle, again only in the placebo group (ρ = −0.55, *P* = 0.014, Figure S4B). In *quadriceps*, an overall significant correlation was observed between baseline PME/γATP (but no other ^31^P MRS indices) and Δ6MWD, when combining both groups (ρ = −0.77, *P* = 0.003, Figure S4C).

Few significant correlations were found between the quantitative MRI or ^31^P MRS indices and clinical variables such as CK. Tables S10 and S11 offer a comprehensive summary of the correlation analyses.

## Discussion

This study demonstrated that muscle FF increased, and cCSA decreased to a greater extent in placebo‐treated compared with sirolimus‐treated IBM patients. Water T_2_, as an MRI‐based quantitative biomarker of active muscle damage, increased with higher FF, indicating that muscle fat replacement and active muscle damage coexisted in IBM. ^31^P MRS indices were abnormal in IBM as found in other neuromuscular disorders. Functional and strength decline correlated with quantitative MRI and ^31^P MRS parameters.

### Extent of disease severity

Similar to previous quantitative MRI studies in IBM, the current work found significant heterogeneity in muscle involvement, especially in the *quadriceps* and *triceps surae*.^19^, ^22^ The extent of muscle involvement in thigh and leg, including a slight asymmetry, aligned with earlier research.^5^, ^16^, ^18^, ^19^, ^22^ Most patients (86%) had higher FF in the thigh than the leg, with stronger involvement of anterior thigh muscles. Around 70–75% of cases had anterior‐dominant thigh involvement and posterior‐dominant leg involvement, confirming previous reports about MRI in IBM.^13^, ^15^, ^16^, ^18^, ^19^, ^22^ *Gastrocnemius medialis* and *quadriceps* muscles were heavily affected, except the *rectus femoris* which was relatively spared, similar to findings from other studies.^14^, ^15^, ^16^, ^18^ A strong correlation was found between the FF values of *rectus femoris* in the thigh and the *gastrocnemius medialis* in the leg, two muscles with a muscle fat replacement pattern of interest in IBM. Other idiopathic inflammatory myopathies such as immune‐mediated necrotizing myopathy, dermatomyositis, and polymyositis are known to be more symmetric, show a more posterior thigh involvement, and demonstrate a generally lower degree of muscle fat replacement as compared with IBM.^30^ In other adult non‐inflammatory neuromuscular disorders, thigh involvement is most often predominantly posterior, such as limb‐girdle muscular dystrophy (LGMD) type R1 or calpainopathy,^31^ GNE myopathy (a form of hereditary inclusion body myopathy and notably also characterized by the presence of rimmed vacuoles)^16^, ^28^ and late‐onset Pompe disease,^26^, ^32^ or mixed anterior–posterior, such as LGMD type R9^26^ and dysferlinopathy.^26^, ^33^

### Active muscle damage

Water T_2_ reflects the muscle water content and is a marker of active muscle damage (such as inflammation, necrosis, and oedema),^17^ and was, in the current study, abnormally high in the thigh, especially in the more affected anterior muscles. This observation contrasts with findings done in other disorders such as GNE myopathy,^28^ late‐onset Pompe disease,^32^ and dysferlinopathy^33^ (the latter known to also have an inflammatory component), where water T_2_ was found to be higher in the muscles with relatively less FF. Also, in Duchenne muscular dystrophy (DMD) water T_2_ was found to be abnormal in normal‐looking muscle, as a precursor for FF increase later on in the disease.^34^ The coexistence of muscle fat replacement and abnormal (water) T_2_ has been shown earlier in non‐quantitative MRI studies in IBM^12^, ^13^, ^15^ and is consistent with the IBM autoimmune hypothesis of chronic inflammation resulting in accumulative muscle damage reflected by the increase in FF.^2^, ^4^

^31^P MRS‐based quantitative biomarkers of active muscle damage were elevated in IBM patients, reflecting possible anomalies in phospholipid membrane metabolism (elevated PDE/γATP and PME/γATP)^17^, ^24^, ^28^, ^33^, ^34^ or dysfunctional glycolysis (elevated PME/γATP).^35^ Additionally, similar to a previous study in IBM,^36^ pH_w_ increased, reflecting ionic disturbances potentially linked to an expanded interstitial/extracellular compartment and sarcolemma damage. Unlike diseases such as DMD,^34^ and dysferlinopathy,^33^ where these changes occurred before muscle destruction, IBM patients already had FF levels consistently above 10% in both *triceps surae* and *quadriceps*, similarly as found in facioscapulohumeral dystrophy^37^ and GNE myopathy,^28^ where muscle fat replacement and metabolic changes occurred simultaneously.

### Effect of sirolimus

Disease progression, measured by an increase in FF, was slower in the sirolimus group, as reported earlier for global thigh and leg segments in these IBM patients.^21^ In untreated patients, there was an annual FF increase of about 3%, along with a cCSA decrease of around 9% in the thigh, aligning with earlier quantitative MRI studies in IBM.^19^, ^22^ The most significant difference in ∆FF between the groups was seen in *vastus lateralis* (3.5% in placebo vs. 0.9% in sirolimus), one of the fastest‐progressing muscles in IBM. In the current study, global thigh FF proved to be the most sensitive MRI‐based quantitative biomarker for detecting 1‐year changes between treated and untreated patients, with *quadriceps* and *vasti lateralis/intermedius* also suitable options. Unlike in the study of Morrow et al.,^19^ here, leg‐related FF values had lower sensitivity (SRM < 0.8) probably due to slower leg disease progression and disease heterogeneity. From the functional and strength outcome measures, only 6MWD was found to be sensitive enough to detect 1‐year changes between treated and untreated patients.^21^

Despite the anti‐inflammatory properties of sirolimus (i.e., CD8^+^ T blood cell levels were significantly lower after sirolimus treatment^21^), water T_2_ was overall increased in the treated group. However, as water T_2_ is a sensitive but non‐specific MRI‐based quantitative biomarker for active muscle damage (and thus not only reflecting inflammation), the observed 1‐year increases in some patients may have been due to other causes, such as oedema. In fact, oedema has proven to be a secondary effect of mTOR inhibitors such as sirolimus.^8^ Indeed, the increase in water T_2_ in the sirolimus group at year‐1 was mainly influenced by a small subset of patients (*n* = 5), all of them with a clinical history of oedema and/or lower limb injuries. Nevertheless, most patients in this study, regardless of their baseline water T_2_, whether in treated or untreated patients, showed rather constant water T_2_ levels between baseline and year‐1 MRI exams, in thigh at least. In leg, however, there was more variation between baseline and year‐1 water T_2_ values for individual patients, with some patients demonstrating an increase and others a decrease, but again, this was observed in both treated and untreated patients. Because water T_2_ reflects and is very sensitive to active muscle damage that may be persistent but can also be acute and transient, it remains difficult to interpret changes between two subsequent exams without further longitudinal assessments, even further complicated by the potential influence of therapeutic intervention.^17^

### Early indicators of disease progression

In IBM patients, there was an overall positive yet weak correlation between baseline water T_2_ levels and increase in FF (or decrease in cCSA), and the most evident in hamstring muscles where muscle fat replacement was far less than in anterior thigh muscles. Similar findings were observed in GNE myopathy,^28^ late‐onset Pompe disease,^32^ and dysferlinopathy,^33^ where elevated water T_2_ values in relatively preserved muscle tissue were considered early indicators of faster disease progression compared with muscles with normal water T_2_. Although we did not find a significant correlation between PME/γATP and 1‐year changes in FF or cCSA in this study, ^31^P MRS indices have shown promising results in relation to changes in quantitative MRI parameters such as FF and cCSA in other studies.^28^, ^33^

### Relationship with function and strength

Similar to earlier studies in IBM, muscle fat replacement (FF) and the amount of residual muscle tissue (cCSA) were strongly related to muscle function and strength,^19^, ^22^ but because all patients remained ambulant, there was no threshold that could be established for 6MWD to distinguish between gain and loss of ambulation. The correlation between greater FF increase (∆FF) and greater 6MWD decline (∆6MWD) was driven by the 1‐year changes found in the placebo group and was described already in Benveniste et al.^21^ Knee extension and knee flexion strength decline in most patients over the course of 1 year and correlated well with FF and cCSA, regardless of the patient group.

The correlations between 6MWD and water T_2_ or PME/γATP as biomarkers of active muscle damage were interesting, as they, like FF and cCSA, are potential indicators of muscle function. These MRI and ^31^P MRS‐based quantitative biomarkers could predict a faster decline in function over 1 year, as recently demonstrated in dysferlinopathy.^38^

### Limitations

This study had several limitations. First, a small sample size, conducted at a single site, limits data generalizability. Second, only 1‐year changes were assessed prohibiting the evaluation of longer‐term dynamics of muscle fat replacement and active muscle damage. Finally, water T_2_ values have been shown to decrease with increasing FF values,^23^, ^39^, ^40^ but in this study, water T_2_ initially increased with increasing fat replacement until high levels of FF due to concurrent presence of active muscle damage and muscle fat replacement in IBM. Nevertheless, the impact of high FF values on water T_2_ should always be considered in quantitative MRI studies.

## Conclusions

This study successfully utilized MRI‐based quantitative biomarkers to demonstrate the efficacy of sirolimus in IBM patients, particularly those related to muscle atrophy and trophicity, namely, FF and cCSA. Biomarkers indicating active muscle damage, such as water T_2_ and ^31^P MRS indices, were found to be abnormal in certain muscles, and significantly correlated, much like FF and cCSA, with changes observed over 1 year in FF, cCSA, and 6MWD. The comprehensive approach combining quantitative MRI and ^31^P MRS with functional and strength evaluations holds promise for evaluating disease progression and treatment effectiveness in IBM.

## Funding

This project received the following grants: Appel à projet 2012 Institut National de la Santé Et de la Recherche Médicale ‐ Direction Générale de l'Offre de Soin (Inserm‐DGOS) ‘Recherche Clinique Translationnelle’ (#A12035DS) and Association Française contre les Myopathies (AFM).

## Conflict of interest

YA reports grants from Sanofi and personal fees from Eli Lilly and Bristol‐Myers Squibb, outside the submitted work. J‐YH reports personal fees from Biogen and Sarepta, outside the submitted work. PGC reports personal fees from Sanofi Genzyme, Sarepta, and Santhera, outside the submitted work. OB reports grants from Sanofi and personal fees from CSL‐Behring, LFB, Novartis, and Ra Pharma, outside the submitted work. All other authors declare no competing interests.

## Supporting information


**Figure S1.** cCSA at baseline and one‐year changes
**Figure S2.** Examples of FF maps (at baseline and year‐1) in thigh and leg of two patients
**Figure S3.** FF trajectories for individual patients in *vastus lateralis*, *rectus femoris*, and *gastrocnemius medialis*
**Figure S4.** Correlations between quantitative MRI or ^31^P MRS biomarkers and functional/strength outcome measures
**Table S1.** Demographic, clinical and functional data in individual patients at baseline
**Tables S2.** Fat fraction: control, baseline and one‐year changes
**Tables S3.** cCSA: control, baseline and one‐year changes
**Tables S4.** Water T_2_: control, baseline and one‐year changes
**Table S5.** ^31^P MRS
**Table S6.** Right–left differences quantitative MRI
**Table S7.** Correlations quantitative MRI
**Table S8.** Correlations ^31^P MRS and quantitative MRI
**Table S9.** Summary of one‐year changes in function and strength measures
**Tables S10.** Correlations quantitative MRI and clinical/functional/strength parameters
**Tables S11.** Correlations ^31^P MRS‐clinical/functional/strength parameters

## Data Availability

Data can be made available by the corresponding author upon reasonable request.
