# Supplementary material for: Effect of sirolimus on muscle in inclusion body myositis observed with magnetic resonance imaging and spectroscopy
Source: J Cachexia Sarcopenia Muscle. 2024 Apr 13;15(3):1108–20. doi: 10.1002/jcsm.13451 (PMC11154752; doi:10.1002/jcsm.13451)
Supplement: Supplementary file 1 — Figure S1. cCSA at baseline and one‐year changes Figure S2. Examples of FF maps (at baseline and year‐1) in thigh and leg of two patients Figure S3. FF trajectories for individual patients in vastus lateralis, rectus femoris, and gastrocnemius medialis Figure S4. Correlations between quantitative MRI or 31P MRS biomarkers and functional/strength outcome measures Table S1. Demographic, clinical and functional data in individual patients at baseline Tables S2. Fat fraction: control, baseline and one‐year changes Tables S3. cCSA: control, baseline and one‐year changes Tables S4. Water T2: control, baseline and one‐year changes Table S5. 31P MRS Table S6. Right–left differences quantitative MRI Table S7. Correlations quantitative MRI Table S8. Correlations 31P MRS and quantitative MRI Table S9. Summary of one‐year changes in function and strength measures Tables S10. Correlations quantitative MRI and clinical/functional/strength parameters Tables S11. Correlations 31P MRS‐clinical/functional/strength parameters [file JCSM-15-1108-s001.docx]

**SUPPORTING INFORMATION**

**Title: Effect of sirolimus on muscle in inclusion body myositis observed with magnetic resonance imaging and spectroscopy**

**Authors:** Harmen Reyngoudt*, Pierre-Yves Baudin, Ericky Caldas de Almeida Araújo, Damien Bachasson, Jean-Marc Boisserie, Kubéraka Mariampillai, Mélanie Annoussamy, Yves Allenbach, Jean-Yves Hogrel, Pierre G. Carlier, Benjamin Marty, Olivier Benveniste

***Corresponding author:** Harmen Reyngoudt

Affiliation: Institute of Myology, Neuromuscular Investigation Center, NMR Laboratory, Paris, France

E-mail address: [h.reyngoudt@institut-myologie.org](mailto:h.reyngoudt@institut-myologie.org)

**Supplementary Text: Normalization cCSA**

Body surface area (BSA) is calculated using the Mosteller formula^1^:

$BSA=\sqrt{\frac{W\times H}{3600}}$

Where BSA is in m^2^, W is weight in kg, and H is height in cm.

In the formula cm is used for height but the result is in square metres.

Then, contractile cross-sectional area (cCSA) values were corrected for BSA as follows:

$$cCSA\left( normalized \right)=\frac{cCSA}{{BSA}^{\frac{2}{3}}}$$

(based on allometric scaling^2^)

References:

^1^Mosteller RD. Simplified calculation of body-surface area. *N Engl J Med* 1987;**317**:1098.

^2^Zoeller RF, Ryan ED, Gordish-Dressman H, Price TB, Seip RL, Angelopoulos TJ, et al. Allometric scaling of isometric biceps strength in adult females and the effect of body mass index. *Eur J Appl Physiol* 2008;**104**:701–710.

**Supplementary Figure 1** Box-and-whisker plots of baseline cCSA and cCSA changes in all regions of interest in controls and patients


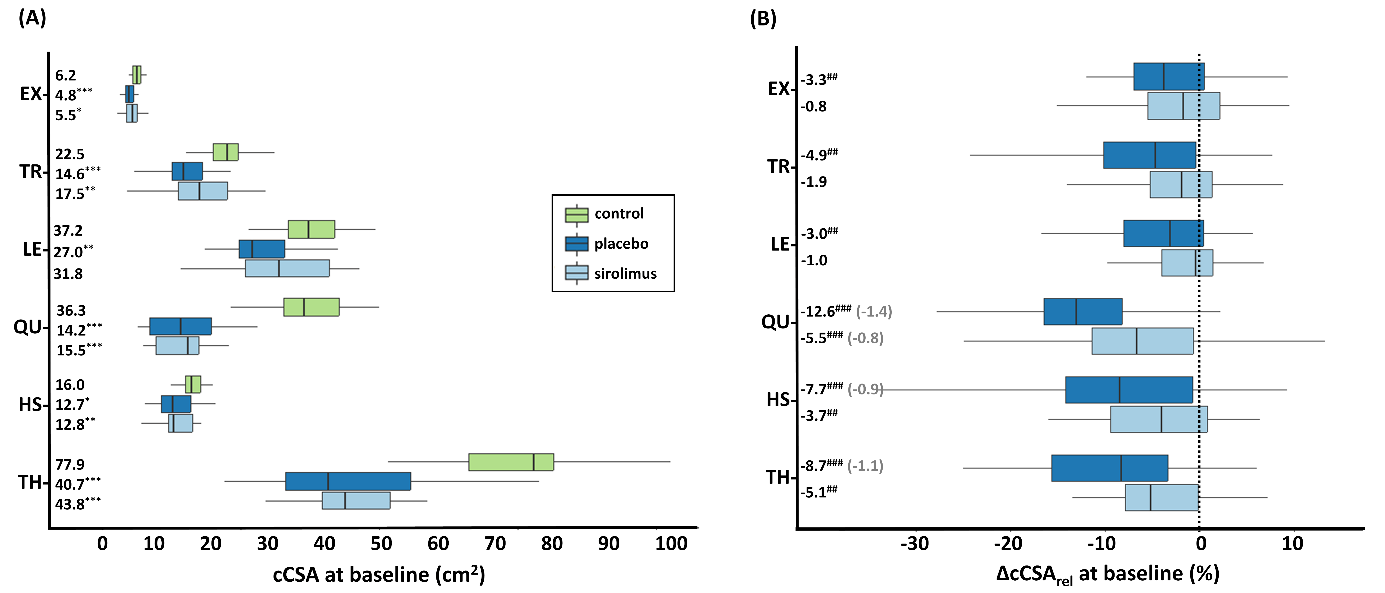


**(A)** Baseline cCSA data in all regions of interest in controls and patients (median values per group are indicated on the left). **(B)** Relative one-year changes in cCSA (median values and SRM values >0.8 per group are indicated on the left). #*P*<0.017; ##*P*<0.01; ###*P*<0.001 (significant differences between baseline and year-1).cCSA, contractile cross-sectional area (cm2); EX, *extensor*; HS, hamstring; LE, global leg; QU, *quadriceps*; TH, global thigh; TR, *triceps surae*; ∆cCSA_rel_, relative 1-year change in cCSA (%).

**Supplementary Figure 2 Examples of FF maps (at baseline and year-1) in thigh and leg of two patients.**


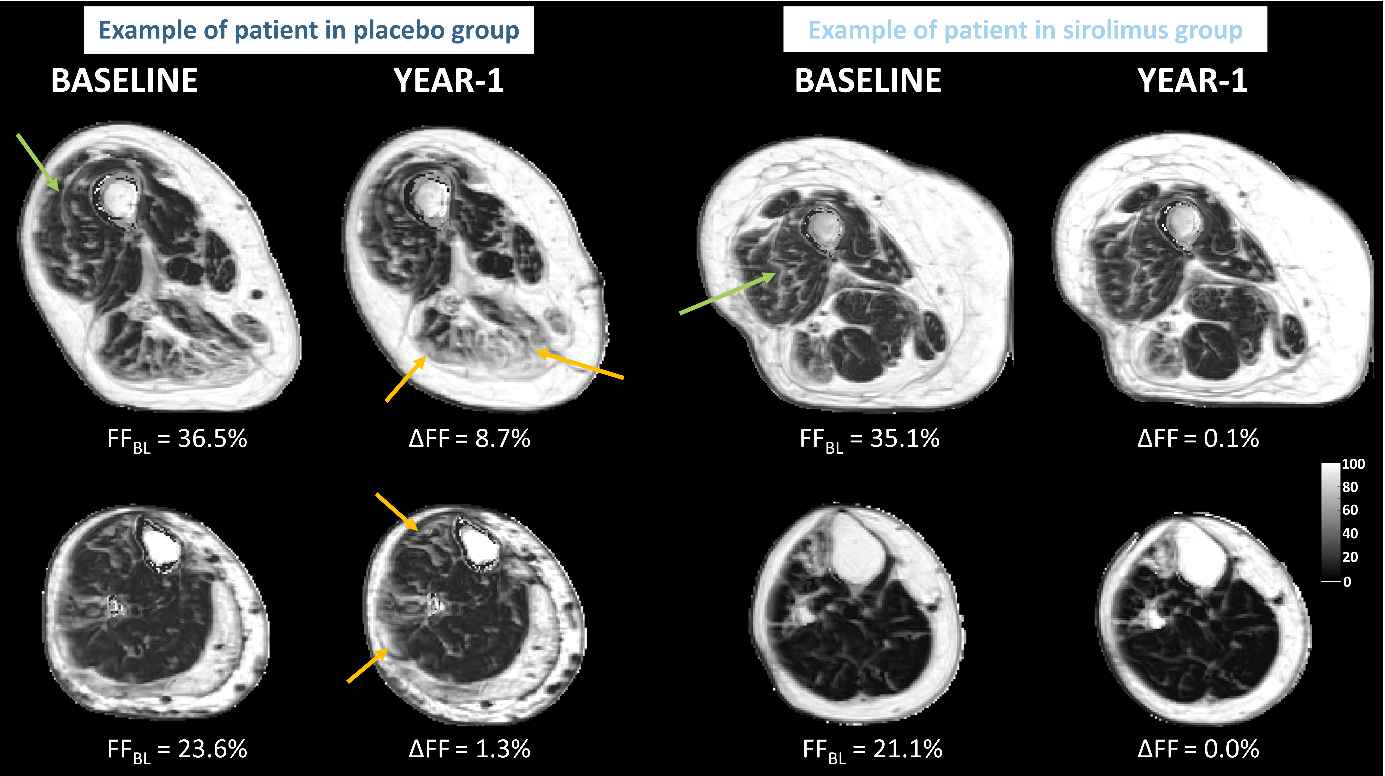


**Images shown here stem from one patient from the placebo group and one selected patient from the sirolimus group with similar baseline FF values. In the patient from the placebo group we observe evident increases in fatty deposits in anterior and posterior thigh compartments and the anterior part of the leg (yellow arrows). In the patient from the sirolimus group, muscle fat replacement did not progress over the course of the study. Notice also the undulating fascia between *vastus intermedius* and *vastus lateralis* in both patients (green arrows), a feature that has been well-described in previous studies in IBM using qualitative high-resolution T_1_-weighted MRI [12,14,15] but were also visible here on the quantitative reconstructed FF maps. FF_BL_, fat fraction at baseline (%); ∆FF, 1-year change in FF (%).**

**Supplementary Figure 3** FF trajectories for individual patients as a function of time since symptom onset

**
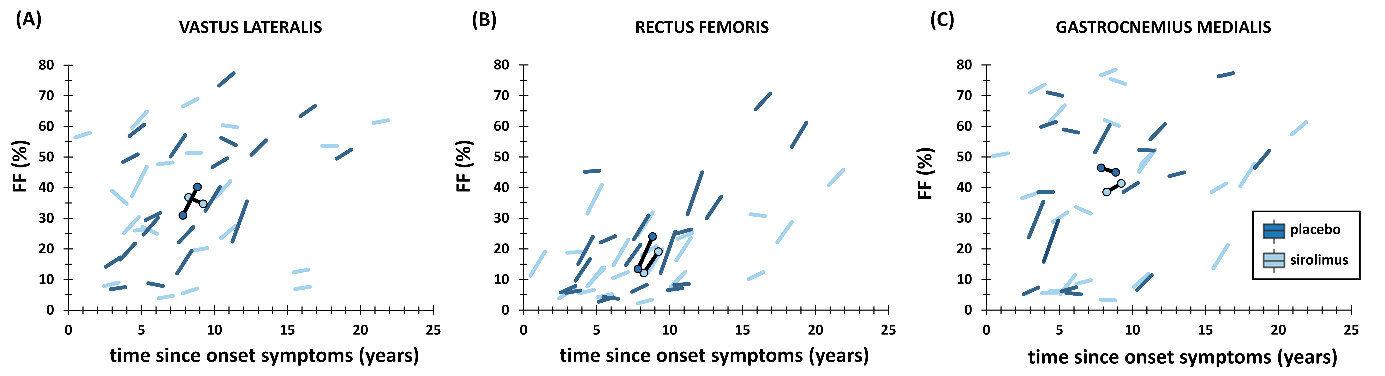
**

**(A)** *vastus lateralis*. **(B)** *rectus femoris*. **(C)** *gastrocnemius medialis*. Median values per group are also depicted in the three plots (black lines). FF, fat fraction (%).

**Supplementary Figure 4** Correlations between quantitative MRI or ^31^P MRS and functional/strength variables

**^
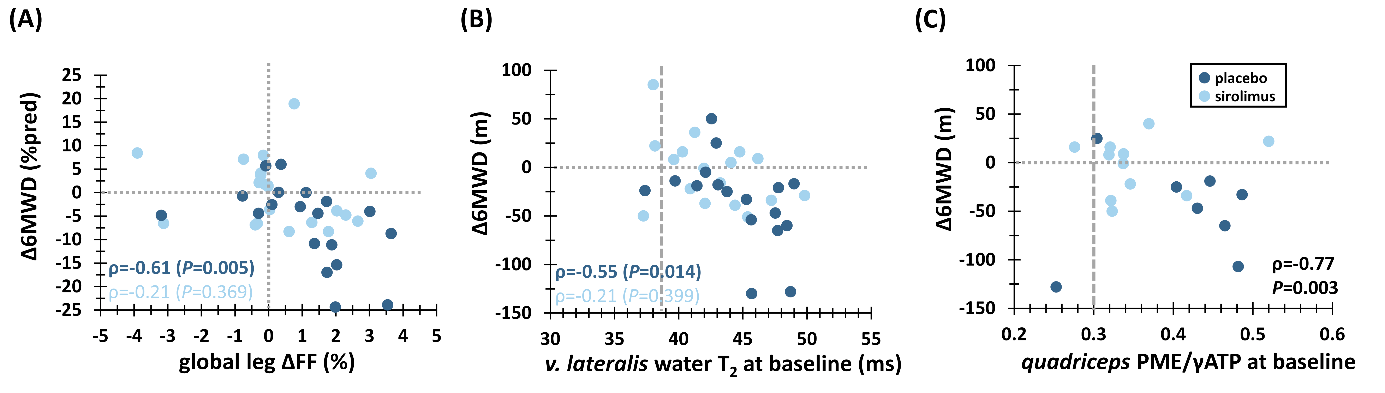
^**

**(A)** Relationship between global leg ∆FF and ∆6MWD (in %pred). **(B)** Relationship between baseline *vastus lateralis* water T_2_ and ∆6MWD (in m). **(C)** Relationship between baseline quadriceps PME/γATP and ∆6MWD (in m). The vertical dashed lines depict the 90^th^ percentile value for *vastus lateralis* water T_2_ (i.e.; 38.9 ms) and and quadriceps PME/γATP (i.e.; 0.30) as determined in controls. Spearman rho (ρ) correlation values and corresponding *P*-values are also indicated for both groups. PME, phosphomonoesters; ATP; adenosine triphosphate (γ refers to γ-resonance of ATP in ^31^P MR spectrum); 6MWD, six-minute-walking distance; ∆FF, one-year change in FF (%); ∆6MWD, one-year change in 6MWD (%pred).

**Supplementary Table 1** Demographic, clinical and functional data in individual patients at baseline

| **Patient n°** | **Group** | **Sex** | **BMI**  **(kg/m^2^)** | **Age**  **(years)** | **Time since symptom onset**  **(years)** | **Time since diagnosis**  **(years)** | **6MWD**  **(m)** | **CK**  **(U/L)** | **^31^P MRS** |
| --- | --- | --- | --- | --- | --- | --- | --- | --- | --- |
| 1 | sirolimus | M | 26.7 | 68.9 | 6.1 | 1.8 | 357 | 304 | BL, TR |
| 2 | placebo | M | 22.7 | 69.6 | 3.6 | 2.1 | 380 | 451 | BL-Y1, QU |
| 3 | placebo | F | 24.7 | 66.9 | 7.0 | 4.0 | 224 | 720 | n/d |
| 5**^a^** | placebo | M | 22.2 | 78.4 | 10.9 | 6.3 | 97 | 218 | n/d |
| 6 | sirolimus | M | 24.3 | 61.0 | 4.4 | 1.0 | 378 | 888 | BL-Y1, QU |
| 7 | sirolimus | M | 30.4 | 68.7 | 4.5 | 1.2 | 393 | 984 | BL-Y1, QU |
| 8 | sirolimus | F | 24.8 | 66.8 | 10.6 | 7.4 | 466 | 293 | BL-Y1, TR |
| 9 | placebo | M | 25.7 | 71.2 | 11.2 | 6.6 | 157 | 1097 | BL-Y1, QU |
| 10 | placebo | M | 19.5 | 58.8 | 10.3 | 8.1 | 322 | 62 | BL-Y1, TR |
| 11 | sirolimus | F | 23.4 | 74.6 | 8.1 | 2.0 | 312 | 167 | BL-Y1, QU |
| 12 | sirolimus | M | 25.8 | 66.7 | 6.2 | 1.0 | 531 | 304 | BL-Y1, QU |
| 13 | placebo | F | 23.2 | 69.8 | 4.2 | 2.8 | 266 | 269 | n/d |
| 14 | placebo | F | 25.6 | 60.8 | 18.4 | 2.8 | 450 | 254 | BL-Y1, TR |
| 15 | sirolimus | M | 22.9 | 74.6 | 17.4 | 9.7 | 392 | 446 | BL-Y1, TR |
| 16 | placebo | F | 26.9 | 75.9 | 5.5 | 0.3 | 357 | 441 | n/d |
| 17 | placebo | F | 22.4 | 62.2 | 5.1 | 4.1 | 264 | 300 | BL-Y1, QU |
| 18 | sirolimus | M | 26.6 | 70.5 | 15.4 | 0.2 | 423 | 583 | BL-Y1, TR |
| 19 | sirolimus | F | 20.5 | 50.2 | 10.5 | 9.1 | 561 | 326 | BL-Y1, QU |
| 20 | placebo | F | 29.1 | 65.4 | 15.9 | 9.5 | 72 | 233 | n/d |
| 21 | sirolimus | M | 26.8 | 63.4 | 0.5 | 5.6 | 372 | 854 | BL-Y1, TR |
| 22 | sirolimus | M | 18.1 | 69.0 | 5.1 | 4.0 | 445 | 195 | BL, TR |
| 23 | placebo | M | 22.6 | 68.7 | 3.9 | 1.3 | 649 | 423 | BL-Y1, QU |
| 24 | sirolimus | F | 34.6 | 60.1 | 8.5 | 1.5 | 181 | 132 | BL-Y1, QU |
| 25 | placebo | M | 29.2 | 66.7 | 7.4 | 1.8 | 311 | 724 | BL-Y1, QU |
| 26 | placebo | F | 22.0 | 61.0 | 12.5 | 9.0 | 372 | 947 | BL-Y1, TR |
| 27 | placebo | M | 30.0 | 54.8 | 5.3 | 2.1 | 287 | 795 | Y1, QU |
| 28 | sirolimus | M | 24.5 | 57.9 | 4.4 | 0.3 | 364 | 722 | BL-Y1, TR |
| 29 | placebo | M | 29.4 | 75.9 | 7.6 | 4.9 | 225 | 298 | BL-Y1, QU |
| 30 | sirolimus | M | 25.9 | 65.8 | 10.1 | 0.2 | 514 | 325 | BL-Y1, TR |
| 31 | sirolimus | M | 22.2 | 75.9 | 15.5 | 3.6 | 575 | 159 | BL-Y1, TR |
| 32 | placebo | F | 24.2 | 51.6 | 10.5 | 3.1 | 257 | 391 | BL-Y1, TR |
| 33 | placebo | M | 24.0 | 76.0 | 2.9 | 0.5 | 423 | 1394 | BL-Y1, TR |
| 34 | placebo | F | 26.7 | 71.7 | 3.7 | 2.7 | 326 | 566 | BL-Y1, TR |
| 35 | placebo | M | 24.5 | 47.5 | 0.7 | 0.4 | 586 | 512 | BL-Y1, QU |
| 36 | sirolimus | F | 23.5 | 67.4 | 2.4 | 0.5 | 342 | 218 | BL-Y1, QU |
| 37 | sirolimus | F | 18.7 | 78.4 | 3.8 | 0.5 | 351 | 171 | BL-Y1, TR |
| 38 | sirolimus | F | 17.7 | 67.6 | 7.8 | 3.1 | 543 | 362 | BL-Y1, TR |
| 39 | sirolimus | F | 25.8 | 50.0 | 7.9 | 2.3 | 212 | 1489 | BL-Y1, TR |
| 40 | placebo | F | 24.7 | 53.5 | 9.9 | 0.4 | 400 | 4459 | BL-Y1, TR |
| 41 | placebo | F | 22.8 | 57.9 | 2.6 | 0.8 | 500 | 460 | BL-Y1, TR |
| 42 | sirolimus | M | 32.4 | 69.8 | 3.0 | 1.9 | 125 | 654 | BL-Y1, QU |
| 43 | sirolimus | M | 23.7 | 73.7 | 20.9 | 7.9 | 126 | 723 | BL-Y1, TR |
| 44 | sirolimus | M | 23.9 | 74.5 | 3.4 | 0.1 | 540 | 217 | Y1, TR |
| 45 | placebo | F | 23.0 | 78.6 | 9.4 | 3.7 | 227 | 110 | BL-Y1, TR |

^a^Patient 5 did not have a year-1 visit and was excluded from the longitudinal analysis in this work. The column heading ‘^31^P MRS’ decribes whether ^31^P MRS data were acquired, at which visits, and in which muscle group. BL, baseline; BMI, body-mass index (in kg/m^2^); CK, creatine kinase concentration (in units per liter); F, female; M, male; n/a, not available; n/d, not determined; QU, *quadriceps*; TR, *triceps surae*; Y1, year-1; 6MWD, 6-minute walking distance (in m).

**Supplementary Tables 2** Fat fraction

**A. Control, baseline FF and ∆FF**

|  | **Controls**  **FF (%)** | **Patients**  **Placebo**  **Baseline FF (%)** | **Patient**  **Sirolimus**  **Baseline FF (%)** | **Patients**  **All**  **Baseline FF (%)** | **Patients**  **Placebo**  **∆FF (%)** | **Patients**  **Sirolimus**  **∆FF (%)** |
| --- | --- | --- | --- | --- | --- | --- |
| ED | 5.1 [4.2-7.0] | 7.5 [5.0-12.3] | 6.4 [5.0-9.7] | 6.9 [5.1-10.7]**^*^** | 0.5 [-0.6-3.7] (0.4) | 1.0 [-0.5-1.0] (0.3) |
| TA | 4.2 [3.0-4.9] | 8.9 [4.7-22.6]**^**^** | 7.6 [4.6-23.5]**^**^** | 7.6 [4.7-22.6]**^***^** | 1.2 [0.0-3.6] (0.7)**^##^** | 1.0 [-0.3-3.3] (0.6) |
| EX | 6.1 [5.3-8.1] | 12.5 [8.4-22.0]**^***^** | 11.1 [8.3-21.9]**^***^** | 11.1 [8.3-21.9]**^***^** | 0.3 [-0.2-1.6] (0.5) | -0.6 [-1.2-1.3] (0.0) |
| TP | 4.5 [3.8-5.0] | 5.8 [4.5-8.1] | 6.2 [4.3-8.8] | 6.1 [4.3-8.4]**^*^** | 0.5 [-0.2-1.1] (0.4) | -0.1 [-0.4-0.3] (0.2) |
| PE | 7.0 [6.1-8.0] | 11.2 [7.1-32.5]**^**^** | 9.4 [7.6-16.3]**^**^** | 10.8 [7.3-19.6]**^***^** | 1.0 [-0.3-2.9] (0.6)**^##^** | 1.0 [-0.6-3.1] (0.4) |
| SO | 4.7 [4.1-6.0] | 6.2 [4.6-15.3] | 6.6 [4.5-15.4] | 6.6 [4.6-15.1]**^*^** | 1.5 [0.0-3.0] (0.7)**^##^** | 0.4 [-0.3-1.3] (0.3) |
| GM | 4.9 [3.1-6.6] | 45.0 [8.9-59.6]**^***^** | 37.5 [7.3-58.4]**^***^** | 39.5 [7.3-59.2]**^***^** | 1.3 [0.0-5.0] (0.6)**^##^** | 2.7 [0.0-5.5] **(0.8)^##^** |
| GL | 4.6 [3.9-5.6] | 7.0 [3.8-39.6] | 5.6 [4.2-19.0] | 6.2 [4.0-27.2] | 1.8 [-0.2-4.1] (0.5)**^##^** | 0.4 [0.0-3.1] (0.5)**^#^** |
| TR | 7.1 [5.4-8.8] | 15.6 [8.5-26.4]**^***^** | 12.6 [9.6-27.3]**^***^** | 13.9 [9.5-26.8]**^***^** | 1.6 [-0.1-2.2] (0.7)**^##^** | 0.2 [-0.6-1.3] (0.3) |
| LE | 7.0 [5.8-8.4] | 20.5 [15.9-34.8]**^***^** | 19.9 [16.1-31.3]**^***^** | 20.5 [16.0-32.3]**^***^** | 1.4 [0.2-2.0] (0.7)**^##^** | 0.0 [-0.3-1.8] (0.2) |
| RF | 2.6 [1.6-3.6] | 12.1 [5.9-22.3]**^***^** | 11.7 [7.7-21.3]**^***^** | 12.1 [6.3-22.1]**^***^** | 2.1 [0.7-7.5] **(0.9)^##^** | 4.9 [1.9-7.4] **(1.6)^###^** |
| VL | 4.0 [3.2-5.8] | 29.4 [13.1-50.4]**^***^** | 37.1 [17.7-54.4]**^***^** | 32.4 [14.1-51.2]**^***^** | 3.5 [2.4-5.4] **(1.1)^###^** | 0.9 [0.1-2.9] (0.5)**^#^** |
| VI | 3.5 [2.9-4.8] | 22.2 [14.3-41.8]**^***^** | 20.5 [14.8-51.5]**^***^** | 20.7 [15.4-46.5]**^***^** | 4.7 [3.0-6.1] **(1.2)^###^** | 2.6 [0.8-5.5] (0.2)**^###^** |
| VM | 2.5 [1.7-3.7] | 22.4 [10.8-40.2]**^***^** | 29.1 [12.5-52.8]**^***^** | 28.3 [11.9-47.9]**^***^** | 3.7 [2.9-5.7] **(1.0)^##^** | 3.0 [1.0-6.0] (0.7)**^##^** |
| QU | 6.7 [4.6-8.2] | 26.8 [19.6-47.8]**^***^** | 29.5 [18.9-51.8]**^***^** | 29.3 [19.5-50.2]**^***^** | 3.2 [1.8-4.8] **(1.3)^###^** | 0.7 [-0.6-3.8] (0.5) |
| AM | 3.6 [2.6-5.1] | 14.2 [6.2-25.8]**^***^** | 16.3 [5.4-39.1]**^***^** | 14.2 [5.9-32.6]**^***^** | 3.7 [1.5-7.4] **(1.0)^###^** | 2.2 [0.1-4.5] (0.6)**^##^** |
| AL | 3.6 [2.0-5.3] | 5.5 [3.6-13.1] | 6.7 [4.2-10.7]**^**^** | 6.6 [3.9-11.5]**^***^** | 2.1 [0.0-4.8] (0.5)**^##^** | 1.6 [-0.5-5.9] (0.5) |
| GR | 5.9 [2.6-9.6] | 7.4 [4.7-19.5] | 9.7 [5.6-22.8] | 9.0 [5.2-19.9] | 4.9 [0.4-8.9] **(0.9)^###^** | 2.5 [-0.5-10.7] (0.6)**^#^** |
| SA | 7.4 [3.9-9.0] | 15.1 [8.4-27.0]**^**^** | 22.0 [12.1-42.5]**^***^** | 19.1 [10.3-35.6]**^***^** | 3.1 [0.8-5.4] (0.1)**^##^** | 0.5 [-1.4-3.3] (0.3) |
| BF | 4.6 [3.2-5.7] | 6.8 [4.5-11.3] | 7.5 [5.5-18.2]**^**^** | 7.4 [5.3-12.5]**^***^** | 0.6 [0.0-1.5] (0.5)**^##^** | 0.3 [-0.3-1.2] (0.3) |
| SM | 5.1 [3.7-7.7] | 13.4 [7.4-57.1]**^***^** | 10.0 [6.2-30.3]**^*^** | 11.6 [7.2-35.0]**^***^** | 2.0 [0.5-4.1] **(0.8)^##^** | 1.5 [0.0-5.0] (0.5)**^##^** |
| ST | 4.8 [3.4-6.0] | 8.6 [7.0-18.3]**^***^** | 12.0 [8.2-26.0]**^***^** | 9.9 [7.2-23.5]**^***^** | 1.5 [0.5-5.4] **(0.8)^###^** | 0.5 [-0.6-5.1] (0.5) |
| HS | 8.6 [6.2-13.7] | 20.8 [13.7-29.3]**^***^** | 20.6 [13.6-25.7]**^***^** | 20.7 [13.7-25.5]**^***^** | 2.2 [-0.3-4.4] (0.7)**^##^** | -0.5 [-1.2-1.9] (0.1) |
| TH | 9.4 [6.5-12.9] | 31.1 [21.2-37.2]**^***^** | 34.8 [22.5-43.0]**^***^** | 31.5 [22.0-39.0]**^***^** | 3.3 [1.4-5.2] **(1.6)^###^** | 1.4 [-0.1-2.9] (0.5)**^#^** |

**P*<0.017; ***P*<0.01; ****P*<0.001 (significant differences between controls and patients). ^#^*P*<0.017; ^##^*P*<0.01; ^###^*P*<0.001 (significant differences between baseline and year-1). All results are indicated with a median value and the inter-quartile distance between square brackets. SRM values are indicated between round brackets. AL, *adductor longus*; AM, *adductor magnus*; BF, *biceps femoris* (long head); ED, *extensor digitorum longus*; EX, *extensor*; FF, fat fraction (%); GL, *gastrocnemius lateralis*; GM, *gastrocnemius medialis*; GR, *gracilis*; HS, hamstring;; LE, global leg; PE, *peroneus longus*; QU, *quadriceps*; SA*, sartorius*; SM, *semimembranosus*; SO, *soleus*; SRM, standardized response mean; ST, *semitendinosus*; TA, *tibialis anterior*; TH, global thigh; TR, *triceps surae*; TP, *tibialis posterior*; VI, *vastus intermedius*; VL, *vastus lateralis*; VM, *vastus medialis*; ∆FF, one-year change in FF (%).

**B. Linear mixed model analysis**

|  | ***β*** | **SE(*β*)** | **95% CI** | ***P*** |
| --- | --- | --- | --- | --- |
| **global segment ∆FF (%)** |  |  |  |  |
| Intercept^a^ | **3.3** | **0.5** | **[2.3 - 4.3]** | **<0.001** |
| Group=Sirolimus | **-1.3** | **0.5** | **[-2.1 - -0.3]** | **0.008** |
| Segment=LE | **-1.8** | **0.5** | **[-2.9 - -0.8]** | **<0.001** |
| **muscle group ∆FF (%)** |  |  |  |  |
| Intercept^b^ | **3.0** | **0.5** | **[2.1 - 3.9]** | **<0.001** |
| Group=Sirolimus | **-1.2** | **0.3** | **[-1.8 - -0.5]** | **<0.001** |
| Muscle=HS | -0.8 | 0.7 | [-2.2 - 0.6] | 0.278 |
| Muscle=TR | **-1.4** | **0.5** | **[-2.4 - -0.3]** | **0.011** |
| Muscle=EX | **-2.0** | **0.5** | **[-3.0 - -1.0]** | **<0.001** |
| **muscle ∆FF (%)** |  |  |  |  |
| Intercept^c^ | **3.0** | **0.5** | **[2.0 - 4.0]** | **<0.001** |
| Group=Sirolimus | **-0.7** | **0.2** | **[-1.1 - -0.2]** | **0.003** |
| Muscle=VM | 1.0 | 0.8 | [-0.5 - 2.6] | 0.195 |
| Muscle=VI | 1.3 | 0.7 | [-0.2 - 2.7] | 0.080 |
| Muscle=RF | 1.7 | 0.8 | [0.2 - 3.3] | 0.031 |
| Muscle=BF | -1.1 | 0.7 | [-2.5 - 0.3] | 0.111 |
| Muscle=SM | 0.3 | 0.9 | [-1.4 - 2.0] | 0.705 |
| Muscle=ST | 0.6 | 0.9 | [-1.3 - 2.4] | 0.540 |
| Muscle=AM | 1.1 | 0.8 | [-0.5 - 2.8] | 0.184 |
| Muscle=AL | 0.7 | 1.1 | [-1.4 - 2.8] | 0.512 |
| Muscle=GR | 2.4 | 1.1 | [0.1 - 4.7] | 0.037 |
| Muscle=SA | -0.5 | 0.8 | [-2.0 - 1.1] | 0.555 |
| Muscle=GM | 0.3 | 0.8 | [-1.2 - 1.8] | 0.714 |
| Muscle=GL | -0.6 | 0.7 | [-2.0 - 0.8] | 0.372 |
| Muscle=SO | -1.2 | 0.6 | [-2.5 - 0.05] | 0.059 |
| Muscle=PE | -1.3 | 0.6 | [-2.5 - 0.02] | 0.053 |
| Muscle=TP | **-2.1** | **0.6** | **[-3.2 - 1.0]** | **<0.001** |
| Muscle=TA | -0.8 | 0.6 | [-2.1 - 0.5] | 0.208 |
| Muscle=EX | -1.8 | 0.6 | [-3.0 - -0.6 | **0.004** |

^a^Estimate (*β*) for global thigh segment in placebo group. ^b^Estimate for *quadriceps* muscle group in placebo group. ^c^Estimate for *vastus lateralis* muscle in placebo group. CI, confidence interval; SE, standard error.

**Supplementary Tables 3** Contractile cross-sectional area

**A. Control, baseline cCSA and ∆cCSA_rel_**

|  | **Controls**  **cCSA**  **(cm^2^)** | **Patients**  **Placebo**  **Baseline cCSA (cm^2^)** | **Patient**  **Sirolimus**  **Baseline cCSA (cm^2^)** | **Patients**  **All**  **Baseline cCSA (cm^2^)** | **Patients**  **Placebo**  **∆cCSA_rel_**  **(%)** | **Patients**  **Sirolimus**  **∆cCSA_rel_**  **(%)** |
| --- | --- | --- | --- | --- | --- | --- |
| EX | 6.2 [5.4-6.9] | 4.8 [4.0-5.7]**^***^** | 5.5 [4.1-6.2]**^*^** | 5.0 [4.1-6.1]**^***^** | -3.3 [-6.0-0.1] (-0.7)**^##^** | -0.8 [-5.7-1.9] (-0.3) |
| TR | 22.5 [19.5-25.0] | 14.6 [12.4-18.1]**^***^** | 17.5 [13.3-23.4]**^**^** | 16.6 [12.6-20.4]**^***^** | -4.9 [-11.1-0.3] (-0.7)**^##^** | -1.9 [-3.9-1.1] (-0.3) |
| LE | 37.2 [33.0-42.2] | 27.0 [23.8-32.8]**^**^** | 31.8 [25.5-41.3] | 30.7 [25.1-38.2]**^**^** | -3.0 [-7.6- -0.4] (-0.7)**^##^** | -1.0 [-3.7-0.8] (-0.3) |
| QU | 36.3 [31.3-43.3] | 14.2 [7.9-21.1]**^***^** | 15.5 [9.4-17.7]**^***^** | 14.5 [9.2-18.0]**^***^** | -12.6 [-17.4- -7.7] **(-1.4)^###^** | -5.5 [-11.6- -1.8] **(-0.8)^###^** |
| HS | 16.0 [14.6-17.7] | 12.7 [10.1-16.2]**^*^** | 12.8 [11.6-16.5]**^**^** | 12.7 [11.2-16.3]**^**^** | -7.7 [-14.5- -2.7] **(-0.9)^###^** | -3.7 [-10.4-0.2] (-0.7)**^##^** |
| TH | 77.9 [63.2-85.8] | 40.7 [32.7-56.0]**^***^** | 43.8 [39.0-52.2]**^***^** | 43.1 [35.5-53.7]**^***^** | -8.7 [-15.3- -3.1] **(-1.1)^###^** | -5.1 [-9.2-0.2] (-0.7)**^##^** |

**P*<0.017; ***P*<0.01; ****P*<0.001 (significant differences between controls and patients). ^#^*P*<0.017; ^##^*P*<0.01; ^###^*P*<0.001 (significant differences between baseline and year-1). All results are indicated with a median value and the inter-quartile distance between square brackets. SRM values are indicated between round brackets. cCSA, cross-sectional area; EX, *extensor*; HS, hamstring; LE, global leg; QU, *quadriceps*; SRM, standardized response mean; TH, global thigh; TR, *triceps surae*; ∆cCSA_rel_, one-year relative change in cCSA (%).

**B. Linear mixed model analysis**

|  | ***β*** | **SE(*β*)** | **95% CI** | ***P*** |
| --- | --- | --- | --- | --- |
| **global segment ∆CSA_rel_ (%)** |  |  |  |  |
| Intercept^a^ | **-8.6** | **1.4** | **[-11.4 - -5.8]** | **<0.001** |
| Group=Sirolimus | 3.3 | 1.5 | [0.4 - 6.2] | 0.026 |
| Segment=LE | **3.7** | **1.5** | **[0.7 - 6.7]** | **0.016** |
| **muscle group ∆CSA_rel_ (%)** |  |  |  |  |
| Intercept^b^ | **-10.9** | **1.4** | **[-13.7 - -8.2]** | **<0.001** |
| Group=Sirolimus | **4.0** | **1.1** | **[1.8 - 6.1]** | **<0.001** |
| Muscle=HS | 1.6 | 1.9 | [-2.2 - 5.3] | 0.410 |
| Muscle=TR | **5.3** | **1.6** | **[2.1 - 8.4]** | **0.001** |
| Muscle=EX | **5.8** | **1.5** | **[2.7 - 8.8]** | **<0.001** |

^a^Estimate (*β*) for global thigh segment in placebo group. ^b^Estimate for *quadriceps* muscle group in placebo group. CI, confidence interval; SE, standard error.

**Supplementary Tables 4** Water T_2_

**A. Control, baseline water T_2_ and ∆T2**

|  | **Controls**  **Water T_2_**  **(ms)** | **Patients**  **Placebo**  **Baseline Water T_2_ (ms)** | **Patient**  **Sirolimus**  **Baseline Water T_2_ (ms)** | **Patients**  **All**  **Baseline Water T_2_ (ms)** | **Patients**  **Placebo**  **∆Water T_2_**  **(ms)** | **Patients**  **Sirolimus**  **∆Water T_2_**  **(ms)** |
| --- | --- | --- | --- | --- | --- | --- |
| ED | 36.5 [35.5-38.1] | 38.2 [37.6-39.5] | 37.4 [36.1-38.6] | 37.8 [36.2-39.2] | -0.1 [-6.5-1.6] (0.1;0.3) | 0.7 [-2.9-2.6] (0.3;0.2) |
| TA | 36.7 [35.9-38.5] | 38.6 [37.4-40.8] | 39.3 [36.5-40.9] | 39.1 [37.4-40.8]**^*^** | -1.4 [-5.1-1.0] (0.1;-0.4) | 0.3 [-4.9-2.2] (0.6;0.3) |
| EX | 36.5 [35.6-38.0] | 39.0 [37.5-39.7]**^**^** | 37.9 [36.3-39.3] | 38.6 [37.3-39.5]**^**^** | -0.5 [-5.4-1.6] (-0.5;-0.3) | 1.4 [-2.5-2.6] (0.1;0.3) |
| TP | 37.1 [36.7-37.9] | 38.8 [37.1-41.6]**^*^** | 38.2 [37.4-40.5] | 38.5 [37.3-40.9]**^*^** | 0.3 [-4.4-1.3] (0.3;0.1) | 1.0 [-3.8-2.1] (0.1;0.4) |
| PE | 37.5 [36.8-38.7] | 39.2 [37.6-39.9] | 37.5 [36.8-39.8] | 38.8 [37.0-39.9] | 0.0 [-5.2-2.1] (0.2;0.1) | 1.1 [-3.9-2.3] (0.1;0.3) |
| SO | 38.0 [36.6-39.3] | 38.6 [36.9-41.1] | 37.0 [36.4-39.3] | 37.8 [36.4-40.7] | 0.8 [-4.4-1.7] (0.2;0.5) | 1.2 [-3.3-2.7] (0.2;0.4) |
| GM | 37.6 [36.9-38.5] | 39.4 [36.8-41.6] | 39.3 [36.7-41.4] | 39.3 [36.9-41.1] | -0.8 [-5.6-0.3] (-0.5;-0.3) | 1.9 [-2.8-3.8] (0.2;0.6) |
| GL | 38.2 [37.4-39.0] | 39.3 [36.7-42.4] | 36.9 [36.0-39.5] | 37.5 [36.1-41.1] | 0.6 [-4.5-2.1] (0.1;0.3) | 0.7 [-3.7-2.8] (0.1;0.3) |
| TR | 37.8 [36.9-39.2] | 38.8 [37.1-41.2] | 37.1 [36.4-39.6] | 37.8 [37.0-40.7] | 1.0 [-3.3-1.7] (0.3;0.2) | 1.0 [-3.3-2.5] (0.2;0.4) |
| LE | 37.6 [36.9-38.7] | 39.2 [37.5-41.0] | 37.3 [36.7-39.4] | 38.3 [37.1-40.7] | 0.8 [-4.2-1.9] (0.3;-0.2) | 1.0 [-2.4-2.2] (0.1;-0.3) |
| RF | 34.1 [32.8-35.5] | 40.4 [37.0-44.7]**^***^** | 40.1 [36.8-42.3]**^***^** | 40.3 [36.9-43.6]**^***^** | 0.3 [-1.4-2.2] (0.1;**3.2**) | 2.1 [0.1-4.1] (**1.1**;**4.0**)**^##^** |
| VL | 36.6 [35.4-37.8] | 45.3 [42.1-47.8]**^***^** | 42.1 [40.3-44.8]**^***^** | 43.5 [41.2-46.4]**^***^** | 0.5 [0.0-1.1] (0.0;**3.1**) | 1.8 [0.7-3.4] (0.6;**2.6**)**^##^** |
| VI | 36.2 [34.9-37.0] | 43.7 [41.5-45.1]**^***^** | 39.7 [38.7-43.3]**^***^** | 42.2 [39.4-44.9]**^***^** | 0.8 [-0.9-2.5] (0.4;**4.6**) | 2.3 [0.6-3.8] (0.7;**2.6**)**^##^** |
| VM | 35.4 [34.5-36.5] | 42.5 [40.4-44.2]**^***^** | 40.2 [39.3-43.7]**^***^** | 42.0 [39.5-43.5]**^***^** | 0.4 [-0.4-2.3] (0.4;**3.6**) | 0.7 [-1.4-3.2] (0.2;**1.5**) |
| QU | 35.9 [34.8-36.7] | 42.3 [40.8-45.0]**^***^** | 39.9 [39.1-42.7]**^***^** | 41.7 [39.5-43.8]**^***^** | 0.6 [-0.2-1.4] (0.0;**3.6**) | 2.2 [0.6-3.0] (0.4;**2.6**)**^##^** |
| AM | 35.5 [34.5-36.1] | 41.4 [37.1-43.0]**^***^** | 38.2 [35.6-39.4]**^***^** | 38.7 [36.8-41.7]**^***^** | 0.1 [-0.8-1.2] (0.1;**2.4**) | 1.5 [-0.2-3.3] (0.6;**1.8**) |
| AL | 34.9 [33.8-36.0] | 37.9 [36.8-39.6]**^***^** | 36.5 [34.8-37.8] | 37.1 [36.1-39.1]**^***^** | 0.1 [-1.3-2.1] (0.1;0.5) | 0.3 [-1.6-2.1] (0.3;**1.0**) |
| GR | 34.2 [32.9-35.0] | 38.8 [36.2-41.0]**^***^** | 36.5 [34.3-38.9]**^*^** | 37.4 [35.1-40.1]**^***^** | -0.6 [-1.4-1.7] (-0.1;**1.3**) | 1.4 [-0.8-2.8] (0.6;**0.9**) |
| SA | 36.1 [34.1-37.2] | 38.4 [36.3-41.5]**^*^** | 36.8 [33.7-40.2] | 37.7 [35.3-41.3]**^*^** | -0.4 [-1.8-2.9] (0.2;**2.5**) | 2.2 [0.8-6.4] (0.5;**1.3**) |
| BF | 36.3 [35.4-37.0] | 38.3 [37.2-41.2]**^***^** | 37.5 [36.4-39.3] | 37.8 [36.8-39.6]**^***^** | 0.8 [-0.5-1.3] (0.2;**3.1**) | 0.8 [0.0-2.3] (0.6;**1.7**) |
| SM | 36.2 [35.2-36.8] | 38.9 [37.0-41.6]**^***^** | 37.2 [36.2-38.7] | 38.2 [36.6-39.5]**^***^** | 0.8 [-0.3-1.1] (0.1;**1.4**) | 1.0 [0.0-2.7] (0.5;**1.5**)**^##^** |
| ST | 35.3 [33.8-35.8] | 38.1 [35.9-40.1]**^***^** | 36.6 [34.9-38.9]**^*^** | 37.9 [35.5-39.1]**^***^** | 0.2 [-0.9-1.5] (0.3;**2.4**) | 0.4 [-1.1-1.8] (0.3;**1.2**) |
| HS | 35.9 [34.8-36.5] | 38.4 [37.0-40.2]**^***^** | 37.6 [35.8-38.4] | 37.8 [36.2-39.1]**^***^** | 0.5 [-0.1-1.5] (0.0;**2.3**)**^#^** | 0.7 [-0.8-2.1] (0.5;**1.5**) |
| TH | 35.7 [34.9-36.6] | 39.6 [38.2-41.2]**^***^** | 37.8 [36.9-38.7]**^***^** | 38.3 [37.2-40.1]**^*^** | 0.4 [-0.5-1.0] (0.4;**5.2**) | 0.7 [0.3-2.8] (0.7;**1.9**)**^##^** |

**P*<0.017; ***P*<0.01; ****P*<0.001 (significant differences between controls and patients). ^#^*P*<0.017; ^##^*P*<0.01; ^###^*P*<0.001 (significant differences between baseline and year-1). All results are indicated with a median value and the inter-quartile distance between square brackets. SRM/SDM values are indicated between round brackets. AL, *adductor longus*; AM, *adductor magnus*; BF, *biceps femoris* (long head); ED, *extensor digitorum longus*; EX, *extensor*; GL, *gastrocnemius lateralis*; GM, *gastrocnemius medialis*; GR, *gracilis*; HS, hamstring; LE, global leg; PE, *peroneus longus*; QU, *quadriceps*; SA, *sartorius*; SDM, standardized difference mean; SM, *semimembranosus*; SO, *soleus*; SRM, standardized response mean; ST, *semitendinosus*; TA, *tibialis anterior*; TH, global thigh; TR, *triceps surae*; TP, *tibialis posterior*; VI, *vastus intermedius*; VL, *vastus lateralis*; VM, *vastus medialis*; ∆T_2_, 1-year change in water T_2_ (ms).

**B. Linear mixed model analysis**

|  | ***β*** | **SE(*β*)** | **95% CI** | ***P*** |
| --- | --- | --- | --- | --- |
| **global segment ∆T_2_ (ms)** |  |  |  |  |
| Intercept^a^ | 0.3 | 0.3 | [-0.4 - 1.0] | 0.389 |
| Group=Sirolimus | **1.2** | **0.5** | **[-0.3 - 2.1]** | **0.010** |
| Segment=LE | -1.2 | 0.6 | [-2.4 - -0.1] | 0.037 |
| **muscle group ∆T_2_ (ms)** |  |  |  |  |
| Intercept^a^ | 0.6 | 0.4 | [-0.2 - 1.5] | 0.136 |
| Group=Sirolimus | **1.3** | **0.4** | **[0.5 - 2.1]** | **0.002** |
| Muscle=HS | -0.4 | 0.5 | [-1.3 - 0.6] | 0.470 |
| Muscle=TR | -1.5 | 0.5 | [-2.7 - -0.2] | 0.023 |
| Muscle=EX | -1.7 | 0.7 | [-3.2 - -0.3] | 0.021 |
| **muscle ∆T_2_ (ms)** |  |  |  |  |
| Intercept^a^ | 0.7 | 0.5 | [-0.3 - 1.7] | 0.181 |
| Group=Sirolimus | **1.3** | **0.2** | **[0.8 - 1.7]** | **<0.001** |
| Muscle=VM | 0.4 | 0.9 | [-1.3 - 2.2] | 0.624 |
| Muscle=VI | 0.5 | 0.6 | [-0.8 - 1.8] | 0.443 |
| Muscle=RF | 0.08 | 0.7 | [-1.3 - 1.4] | 0.907 |
| Muscle=BF | -0.5 | 0.6 | [-1.7 - 0.7] | 0.433 |
| Muscle=SM | -0.04 | 0.6 | [-1.3 - 1.2] | 0.952 |
| Muscle=ST | -0.7 | 0.6 | [-1.9 - 0.4] | 0.214 |
| Muscle=AM | -0.3 | 0.6 | [-1.5 - 0.8] | 0.568 |
| Muscle=AL | -1.2 | 0.7 | [-2.6 - 0.1] | 0.079 |
| Muscle=GR | -0.8 | 0.6 | [-2.1 - 0.5] | 0.202 |
| Muscle=SA | 0.5 | 0.8 | [-1.1 - 2.0] | 0.563 |
| Muscle=GM | -1.6 | 1.0 | [-3.6 - 0.4] | 0.109 |
| Muscle=GL | -1.5 | 0.9 | [-3.3 - 0.2] | 0.077 |
| Muscle=SO | -1.5 | 0.7 | [-2.9 - -0.1] | 0.034 |
| Muscle=PE | -1.8 | 0.7 | [-3.2 - -0.3] | 0.019 |
| Muscle=TP | **-1.8** | **0.7** | **[-3.3 - -0.4]** | **0.015** |
| Muscle=TA | **-3.0** | **0.8** | **[-4.6 - -1.4]** | **<0.001** |
| Muscle=EX | **-1.9** | **0.8** | **[-3.5 - -0.4]** | **0.015** |

^a^Estimate (*β*) for global thigh segment in placebo group. ^b^Estimate for *quadriceps* muscle group in placebo group. ^c^Estimate for *vastus lateralis* muscle in placebo group. CI, confidence interval; SE, standard error.

**Supplementary Table 5** ^31^P MRS

|  | **CONTROLS** | **PATIENTS**  **BASELINE** | ***P^a^*** |  | **PATIENTS**  **PLACEBO-Y1** | **PATIENTS**  **SIROLIMUS-Y1** | ***P^b^*** |
| --- | --- | --- | --- | --- | --- | --- | --- |
| **TRICEPS SURAE** |  |  |  |  |  |  |  |
| ***n*** | 9 | 22 |  |  | 9 | 11 |  |
| **P_i,tot_/PCr** | **0.14 [0.14-0.15]** | **0.16 [0.15-0.19]** | **<0.001** |  | **0.19 [0.18-0.22]** | **0.16 [0.14-0.18]** | **0.003** |
| **P_i,tot_/γATP** | **0.68 [0.64-0.70]** | **0.76 [0.71-0.83]** | **<0.001** |  | **0.82 [0.80-0.93]** | **0.73 [0.65-0.81]** | **0.005** |
| **PCr/γATP** | 4.76 [4.55-5.02] | 4.45 [4.30-4.74] | 0.099 |  | 4.20 [4.16-4.45] | 4.70 [4.45-4.84] | 0.008 |
| **PDE/γATP** | **0.23 [0.21-0.27]** | **0.43 [0.35-0.46]** | **<0.001** |  | 0.41 [0.34-0.43] | 0.43 [0.36-0.49] | 0.260 |
| **PME/γATP** | **0.19 [0.13-0.24]** | **0.36 [0.32-0.44]** | **<0.001** |  | 0.39 [0.33-0.43] | 0.32 [0.28-0.39] | 0.050 |
| **P_i,b_/P_i,tot_** | 0.12 [0.06-0.19] | 0.13 [0.11-0.14] | 0.489 |  | 0.13 [0.10-0.16] | 0.13 [0.10-0.15] | 0.360 |
| **pH_w_** | 7.05 [7.00-7.09] | 7.08 [7.07-7.11] | 0.015 |  | 7.10 [7.09-7.13] | 7.11 [7.09-7.12] | 0.496 |
| **[Mg^2+^] (mM)** | **0.49 [0.47-0.53]** | **0.44 [0.41-0.46]** | **<0.001** |  | 0.42 [0.40-0.49] | 0.46 [0.41-0.50] | 0.641 |
| **FF (%)** | **7.1 [5.4-8.8]** | **12.5 [9.1-19.4]** | **<0.001** |  | 18.4 [11.5-19.9] | 11.8 [8.9-27.1] | 0.431 |
| **cCSA (cm^2^)** | **32.8 [29.4-39.0]** | **25.8 [18.4-33.0]** | **0.002** |  | 19.2 [17.2-26.2] | 26.2 [18.8-35.4] | 0.096 |
| **water T_2_ (ms)** | 37.8 [36.9-38.9] | 37.5 [36.7-39.6] | 0.938 |  | 39.5 [36.7-41.6] | 38.4 [35.0-40.3] | 0.554 |
|  |  |  |  |  |  |  |  |
| **QUADRICEPS** |  |  |  |  |  |  |  |
| ***n*** | 9 | 15 |  |  | 7 | 8 |  |
| **P_i,tot_/PCr** | 0.12 [0.12-0.14] | 0.16 [0.13-0.23] | 0.023 |  | 0.18 [0.12-0.30] | 0.16 [0.15-0.22] | 0.993 |
| **P_i,tot_/γATP** | 0.66 [0.63-0.71] | 0.69 [0.62-0.79] | 0.431 |  | 0.65 [0.58-0.99] | 0.69 [0.63-0.81] | 0.830 |
| **PCr/γATP** | 5.28 [4.97-5.50]^c^ | 3.46 [4.26-4.69] | 0.014 |  | 4.34 [3.25-4.90] | 4.22 [3.43-4.96] | 0.893 |
| **PDE/γATP** | 0.31 [0.28-0.45] | 0.48 [0.38-0.59] | 0.018 |  | 0.49 [0.34-0.73] | 0.42 [0.38-0.66] | 0.867 |
| **PME/γATP** | **0.25 [0.22-0.28]** | **0.36 [0.33-0.61]** | **<0.001** |  | 0.39 [0.36-0.90] | 0.52 [0.38-0.68] | 0.985 |
| **P_i,b_/P_i,tot_** | **0.08 [0.07-0.11]** | **0.19 [0.14-0.27]** | **0.002** |  | 0.16 [0.12-0.23] | 0.21 [0.14-0.23] | 0.448 |
| **pH_w_** | **7.08 [7.07-7.09]** | **7.13 [7.09-7.17]** | **0.002** |  | 7.20 [7.12-7.25] | 7.15 [7.13-7.24] | 0.913 |
| **[Mg^2+^] (mM)** | **0.51 [0.49-0.57]** | **0.45 [0.39-0.48]** | **0.003** |  | 0.47 [0.40-0.63] | 0.45 [0.39-0.50] | 0.456 |
| **FF (%)** | **6.5 [4.5-8.3]** | **20.6 [14.1-25.8]** | **<0.001** |  | 22.6 [11.0-28.6] | 23.8 [15.7-32.6] | 0.710 |
| **cCSA (cm^2^)** | **55.4 [42.2-70.0]^c^** | **27.9 [21.4-43.5]** | **<0.001** |  | 30.5 [20.2-38.0] | 20.5 [16.3-34.8] | 0.318 |
| **water T_2_ (ms)** | **35.8 [34.8-36.7]^c^** | **40.1 [39.0-43.2]** | **<0.001** |  | 40.1 [39.8-44.7] | 38.8 [38.2-49.4] | 0.731 |

^a^Difference between controls and patient. ^b^Difference between placebo and sirolimus groups at year-1. All results are indicated with a median value and the inter-quartile distance between square brackets. ^c^In controls, PCr/γATP (*P*=0.007), and cCSA (*P*<0.001) were significantly higher in quadriceps as compared to *triceps surae*; water T_2_ was significantly lower in quadriceps compared to *triceps surae* (*P*<0.001). cCSA, contractile cross-sectional area (cm^2^); FF, fat fraction (%); n, number of subjects; PCr, phosphocreatine; PDE, phosphodiesters; pH_w_, weighted pH; P_i,b_, alkaline inorganic phosphate; P_i,tot_, total inorganic phosphate; PME, phosphomonesters; γATP, adenosine triphosphate (γ-resonance in ^31^P MR spectrum); [Mg^2+^], intramuscular magnesium concentration (mM).

**Supplementary Table 6** Right-left differences quantitative MRI

|  | **Left FF**  **(%)** | **Right FF**  **(%)** | **Left water T_2_ (ms)** | **Right water T_2_ (ms)** | **Left cCSA**  **(cm^2^)** | **Right cCSA**  **(cm^2^)** |
| --- | --- | --- | --- | --- | --- | --- |
| ED | 7.1 [5.1-10.2] | 7.0 [5.0-9.6] | 37.4 [36.2-39.1] | 37.9 [35.8-38.4] | - | - |
| TA | 8.3 [4.8-24.2] | 6.5 [4.7-21.4] | 38.2 [36.2-39.8] | 38.9 [37.3-40.9] | - | - |
| EX | 11.0 [7.7-21.5] | 11.5 [8.8-19.8] | 38.4 [36.9-39.6] | 38.3 [36.8-39.4] | 5.0 [3.7-6.1] | 5.1 [4.1-6.0] |
| TP | **6.0 [4.2-7.1]^**^** | **6.1 [4.5-9.4]^**^** | 38.5 [37.0-40.5] | 38.8 [37.4-40.8] | - | - |
| PE | 10.3 [7.1-17.8] | 10.7 [7.3-28.8] | 38.6 [36.7-40.3] | 37.9 [36.7-39.4] | - | - |
| SO | **5.3 [4.0-12.4]^***^** | **6.9 [5.0-16.4]^***^** | 38.3 [36.8-40.6] | 38.0 [36.4-41.1] | - | - |
| GM | **37.3 [5.8-57.3]^**^** | **40.5 [8.7-63.6]^**^** | 39.0 [35.9-41.3] | 39.6 [37.5-42.1] | - | - |
| GL | **6.4 [3.5-34.7]^***^** | **6.7 [4.7-26.6]^***^** | 36.9 [36.0-40.5] | 37.0 [35.9-40.0] | - | - |
| TR | **12.5 [8.4-29.2]^*^** | **14.0 [10.5-25.2]^*^** | 38.1 [37.0-40.1] | 37.9 [36.6-41.3] | 16.4 [13.0-19.6] | 16.4 [12.3-20.3] |
| LE | 19.9 [15.8-31.9] | 20.3 [15.9-35.7] | 38.5 [37.1-40.6] | 38.2 [36.9-40.8] | 29.6 [24.7-37.7] | 31.0 [24.7-37.3] |
| RF | **11.5 [4.8-23.6]^***^** | **15.5 [7.0-26.5]^***^** | 38.9 [35.7-41.3] | 39.5 [37.2-45.5] | - | - |
| VL | **29.2 [12.1-51.1]^***^** | **37.8 [16.0-55.5]^***^** | 42.9 [41.0-46.5] | 42.3 [40.5-45.2] | - | - |
| VI | 20.1 [13.6-47.8] | 25.4 [13.4-45.3] | 43.5 [40.5-45.5] | 41.8 [39.1-44.3] | - | - |
| VM | 29.2 [9.8-48.4] | 24.0 [12.0-46.3] | 40.9 [38.4-43.1] | 41.7 [39.0-44.3] | - | - |
| QU | **27.1 [18.4-49.3]^***^** | **32.4 [20.6-51.1]^***^** | 41.2 [39.2-43.1] | 42.4 [39.5-45.2] | 14.1 [9.7-18.2] | 14.3 [8.9-18.0] |
| AM | 12.4 [5.4-35.0] | 10.2 [5.4-29.7] | 38.6 [36.7-41.8] | 38.6 [37.0-41.7] | - | - |
| AL | 6.0 [3.9-10.5] | 6.5 [3.1-13.0] | **36.2 [34.9-38.0]^**^** | **37.8 [36.2-39.4]^**^** | - | - |
| GR | 10.6 [4.3-21.4] | 9.1 [4.9-22.6] | 36.7 [35.2-39.6] | 36.5 [33.9-39.2] | - | - |
| SA | 18.5 [10.6-32.1] | 17.1 [8.9-38.1] | **35.7 [34.1-39.8]^***^** | **38.5 [35.1-42.1]^***^** | - | - |
| BF | **7.1 [4.4-8.9]^**^** | **7.3 [5.1-16.7]^**^** | 38.1 [37.1-39.9] | 37.9 [36.4-39.7] | - | - |
| SM | **9.8 [7.0-33.8]^**^** | **13.9 [6.7-40.1]^**^** | **39.6 [38.1-40.8]^***^** | **37.4 [35.8-38.6]^***^** | - | - |
| ST | 11.3 [6.8-21.9] | 9.5 [7.2-26.5] | **37.7 [35.7-40.1]^***^** | **36.9 [34.5-39.0]^***^** | - | - |
| HS | 20.7 [13.8-25.7] | 19.7 [14.1-27.7] | 38.0 [36.7-39.4] | 37.5 [35.5-39.0] | 13.2 [11.0-15.5] | 12.9 [11.0-16.3] |
| TH | 29.9 [20.9-38.8] | 32.3 [22.7-39.5] | 38.5 [37.4-39.9] | 38.3 [36.7-40.1] | 43.4 [34.9-52.5] | 41.4 [34.8-52.4] |

**P*<0.017; ***P*<0.01; ****P*<0.001 (significant differences between left and right). Significant differences are indicated in bold. All results are indicated with a median value and the inter-quartile distance between square brackets. AL, *adductor longus*; AM, *adductor magnus*; BF, *biceps femoris* (long head); cCSA, contractile cross-sectional area (cm^2^); ED, *extensor digitorum longus*; EX, *extensor*; FF, fat fraction (%); GL, *gastrocnemius lateralis*; GM, *gastrocnemius medialis*; GR, *gracilis*; HS, hamstring; LE, global leg; PE, *peroneus longus*; QU, *quadriceps*; SA, *sartorius*; SM, *semimembranosus*; SO, soleus; ST, semitendinosus; TA, tibialis anterior; TH, global thigh; TR, triceps surae; TP, tibialis posterior; VI, *vastus intermedius*; VL, *vastus lateralis*; VM, *vastus medialis*; “-“, not assessed

**Supplementary Table 7** Correlations quantitative MRI

|  | **Correlation**  **water T_2_ at baseline - FF at**  **baseline**  **placebo** | | **Correlation**  **water T_2_ at baseline - FF at**  **baseline**  **sirolimus** | | **Correlation**  **water T_2_ at baseline - ∆FF**  **placebo** | | **Correlation**  **water T_2_ at baseline - ∆FF**  **sirolimus** | | **Correlation**  **water T_2_ at baseline - ∆cCSA_rel_ placebo** | | **Correlation**  **water T_2_ at baseline - ∆cCSA_rel_ sirolimus** | |
| --- | --- | --- | --- | --- | --- | --- | --- | --- | --- | --- | --- | --- |
|  | *ρ* | *P* | *ρ* | *P* | *ρ* | *P* | *ρ* | *P* | *ρ* | *P* | *ρ* | *P* |
| ED | **0.71** | **<0.001** | 0.47 | 0.030 | 0.34 | 0.171 | 0.09 | 0.703 | - | - | - | - |
| TA | 0.16 | 0.531 | 0.42 | 0.064 | 0.05 | 0.852 | 0.17 | 0.304 | - | - | - | - |
| EX | 0.41 | 0.078 | 0.32 | 0.169 | 0.45 | 0.056 | 0.39 | 0.091 | -0.19 | 0.433 | -0.43 | 0.139 |
| TP | 0.49 | 0.034 | **0.76** | **<0.001** | 0.001 | 0.997 | 0.17 | 0.475 | - | - | - | - |
| PE | 0.39 | 0.119 | 0.49 | 0.036 | 0.57 | 0.021 | 0.24 | 0.325 | - | - | - | - |
| SO | 0.45 | 0.055 | 0.28 | 0.225 | 0.45 | 0.063 | 0.16 | 0.510 | - | - | - | - |
| GM | -0.09 | 0.762 | 0.46 | 0.064 | 0.20 | 0.542 | 0.55 | 0.022 | - | - | - | - |
| GL | 0.41 | 0.119 | **0.77** | **0.003** | 0.13 | 0.648 | 0.14 | 0.606 | - | - | - | - |
| TR | 0.43 | 0.066 | 0.32 | 0.180 | 0.37 | 0.115 | 0.13 | 0.601 | -0.19 | 0.455 | -0.35 | 0.150 |
| LE | **0.59** | **0.007** | **0.53** | **0.016** | 0.23 | 0.323 | 0.05 | 0.830 | 0.04 | 0.880 | -0.43 | 0.057 |
| RF | **0.62** | **0.006** | 0.31 | 0.177 | **0.64** | **0.008** | 0.26 | 0.274 | - | - | - | - |
| VL | 0.44 | 0.061 | 0.04 | 0.870 | 0.42 | 0.081 | 0.07 | 0.770 | - | - | - | - |
| VI | 0.44 | 0.052 | 0.12 | 0.614 | -0.13 | 0.610 | 0.26 | 0.265 | - | - | - | - |
| VM | 0.12 | 0.618 | 0.23 | 0.349 | 0.27 | 0.263 | 0.40 | 0.103 | - | - | - | - |
| QU | 0.43 | 0.053 | 0.08 | 0.733 | **0.51** | **0.017** | 0.26 | 0.256 | -0.34 | 0.148 | -0.45 | 0.040 |
| AM | **0.72** | **<0.001** | 0.11 | 0.642 | **0.65** | **0.002** | 0.001 | 0.973 | - | - | - | - |
| AL | 0.48 | 0.037 | 0.40 | 0.064 | 0.36 | 0.147 | 0.11 | 0.640 | - | - | - | - |
| GR | **0.71** | **<0.001** | 0.35 | 0.125 | 0.24 | 0.310 | 0.29 | 0.205 | - | - | - | - |
| SA | 0.43 | 0.152 | 0.26 | 0.319 | -0.15 | 0.573 | 0.24 | 0.426 | - | - | - | - |
| BF | 0.38 | 0.086 | 0.47 | 0.032 | **0.70** | **0.001** | 0.23 | 0.311 | - | - | - | - |
| SM | **0.64** | **0.004** | **0.56** | **0.007** | 0.43 | 0.088 | **0.61** | **0.002** | - | - | - | - |
| ST | 0.45 | 0.040 | **0.66** | **<0.001** | **0.70** | **0.001** | **0.68** | **<0.001** | - | - | - | - |
| HS | 0.47 | 0.032 | 0.50 | 0.021 | 0.43 | 0.053 | **0.54** | **0.011** | **-0.53** | **0.016** | **-0.55** | **0.010** |
| TH | 0.14 | 0.552 | -0.30 | 0.207 | 0.40 | 0.084 | 0.10 | 0.677 | **-0.55** | **0.012** | **-0.64** | **0.002** |

Significant differences are indicated in bold (moderate correlations in **light grey**, strong correlations in **dark grey**). AL, *adductor longus*; AM, *adductor magnus*; BF, *biceps femoris* (long head); ED, *extensor digitorum longus*; EX, *extensor*; FF, fat fraction (%); GL, *gastrocnemius lateralis*; GM, *gastrocnemius medialis*; GR, *gracilis*; HS, hamstring; LE, global leg; PE, *peroneus longus*; QU, *quadriceps*; SA, *sartorius*; SM, *semimembranosus*; SO, *soleus*; ST, *semitendinosus*; TA, *tibialis anterior*; TH, global thigh; TR, *triceps surae*; TP*, tibialis posterior*; VI, *vastus intermedius*; VL, *vastus lateralis*; VM, *vastus medialis*; ρ, Spearman-rank correlation coefficient; ∆FF, one-year change in FF (%); ∆cCSA_rel_, one-year relative change in cCSA (%);“-“, not assessed

**Supplementary Table 8** Correlations ^31^P MRS and quantitative MRI

|  | **Correlation with**  **FF (baseline)** | | **Correlation with**  **water T_2_ (baseline)** | | **Correlation**  **with cCSA (baseline)** | | **Correlation with**  **∆FF** | | **Correlation with**  **∆cCSA_rel_** | |
| --- | --- | --- | --- | --- | --- | --- | --- | --- | --- | --- |
|  | *ρ* | *P* | *ρ* | *P* | *ρ* | *P* | *ρ* | *P* | *ρ* | *P* |
| **TRICEPS SURAE** |  |  |  |  |  |  |  |  |  |  |
| P_i,tot_/PCr | 0.44 | 0.037 | 0.34 | 0.128 | -0.48 | 0.022 | 0.06 | 0.792 | -0.16 | 0.481 |
| P_i,tot_/γATP | 0.31 | 0.147 | 0.50 | 0.017 | -0.46 | 0.026 | 0.19 | 0.396 | -0.34 | 0.124 |
| PCr/γATP | -0.44 | 0.038 | -0.12 | 0.597 | 0.38 | 0.070 | 0.02 | 0.981 | -0.09 | 0.706 |
| PDE/γATP | 0.09 | 0.673 | -0.12 | 0.584 | 0.33 | 0.125 | -0.01 | 0.968 | -0.38 | 0.082 |
| PME/γATP | 0.53 | 0.010 | 0.28 | 0.206 | -0.50 | 0.014 | 0.34 | 0.115 | -0.44 | 0.039 |
| P_i,b_/P_i,tot_ | 0.09 | 0.697 | -0.19 | 0.393 | 0.13 | 0.550 | 0.03 | 0.833 | 0.27 | 0.232 |
| pH_w_ | 0.18 | 0.419 | 0.20 | 0.382 | 0.12 | 0.590 | -0.08 | 0.703 | -0.02 | 0.930 |
| [Mg^2+^] (mM) | -0.51 | 0.012 | -0.34 | 0.124 | 0.41 | 0.053 | -0.11 | 0.631 | 0.15 | 0.500 |
| **QUADRICEPS** |  |  |  |  |  |  |  |  |  |  |
| P_i,tot_/PCr | 0.48 | 0.094 | **0.84** | **<0.001** | -0.06 | 0.859 | 0.51 | 0.074 | -0.62 | 0.025 |
| P_i,tot_/γATP | 0.47 | 0.108 | **0.85** | **<0.001** | 0.13 | 0.668 | 0.59 | 0.033 | -0.65 | 0.017 |
| PCr/γATP | -0.45 | 0.122 | -0.68 | 0.010 | 0.32 | 0.280 | -0.37 | 0.209 | 0.58 | 0.039 |
| PDE/γATP | 0.32 | 0.289 | 0.40 | 0.174 | 0.18 | 0.553 | 0.26 | 0.384 | -0.18 | 0.566 |
| PME/γATP | 0.39 | 0.194 | **0.76** | **0.002** | -0.13 | 0.681 | 0.65 | 0.017 | -0.60 | 0.031 |
| P_i,b_/P_i,tot_ | 0.22 | 0.471 | 0.35 | 0.247 | -0.38 | 0.201 | 0.33 | 0.271 | -0.23 | 0.459 |
| pH_w_ | 0.07 | 0.817 | 0.35 | 0.247 | -0.05 | 0.873 | 0.31 | 0.297 | -0.29 | 0.334 |
| [Mg^2+^] (mM) | 0.31 | 0.456 | 0.29 | 0.493 | 0.12 | 0.779 | 0.38 | 0.352 | -0.19 | 0.651 |

Significant differences are indicated in bold (strong correlations in **dark grey**). cCSA, contractile cross-sectional area (cm^2^); FF, fat fraction (%); PCr, phosphocreatine; PDE, phosphodiesters; pH_w_, weighted pH; P_i,b_, alkaline inorganic phosphate; P_i,tot_, total inorganic phosphate; PME, phosphomonesters; γATP, adenosine triphosphate (γ-resonance in ^31^P MR spectrum); [Mg^2+^], intramuscular magnesium concentration (mM); ρ, Spearman-rank correlation coefficient; ∆cCSA_rel_, one-year relative change in cCSA (%); ∆FF, one-year change in FF (%).

**Supplementary Table 9** Summary of one-year changes in function and strength measures

|  | **SRM placebo** | **SRM sirolimus** |
| --- | --- | --- |
| **∆6MWD (m)** | **-0.8** (-36.6/45.3) | -0.1 (-4.1/35.7) |
| **∆6MWD (%pred)** | -**0.8** (-6.6/8.4) | -0.003 (-0.02/7.1) |
| **∆knee extension (Nm)** | -0.7 (-5.0/7.2) | -0.4 (-2.3/5.2) |
| **∆knee extension (%pred)** | -0.6 (-3.2/5.4) | -0.4 (-1.3/3.6) |
| **∆knee flexion (Nm)** | -**1.1** (-6.2/5.8) | **-0.8** (-3.7/4.4) |
| **∆knee flexion (%pred)** | -**0.9** (-8.0/8.7) | **-0.8** (-4.8/6.2) |

High SRM values (i.e., ≥0.8) are indicated in bold. SRM values are calculated as mean divided by standard deviation of ∆ value. A detailed report of these muscle function and strength results in the RAPAMI study can be found in the earlier published paper Benveniste et al.^20^

SRM, standardized response mean; 6MWD, six-minute walking distance.

**Supplementary Tables 10** Correlations quantitative MRI and clinical/functional/strength parameters

**A. Clinical parameters**

|  | **Correlation with**  **Age at BL**  **placebo** | | **Correlation with**  **Age at BL**  **sirolimus** | | **Correlation with**  **#years since symptom onset at BL**  **placebo** | | **Correlation with**  **#years since symptom onset at BL**  **sirolimus** | | **Correlation with**  **BMI at BL**  **placebo** | | **Correlation with**  **BMI at BL**  **sirolimus** | | **Correlation with**  **CK at BL**  **placebo** | | **Correlation with**  **CK at BL**  **sirolimus** | |
| --- | --- | --- | --- | --- | --- | --- | --- | --- | --- | --- | --- | --- | --- | --- | --- | --- |
|  | *ρ* | *P* | *ρ* | *P* | *ρ* | *P* | *ρ* | *P* | *ρ* | *P* | *ρ* | *P* | *ρ* | *P* | *ρ* | *P* |
| **EX** |  |  |  |  |  |  |  |  |  |  |  |  |  |  |  |  |
| FF | 0.17 | 0.470 | 0.12 | 0.584 | 0.22 | 0.352 | 0.15 | 0.510 | 0.38 | 0.099 | 0.23 | 0.301 | 0.02 | 0.950 | -0.05 | 0.813 |
| water T_2_ | 0.24 | 0.322 | 0.16 | 0.154 | 0.11 | 0.668 | 0.16 | 0.502 | 0.40 | 0.099 | 0.41 | 0.073 | 0.08 | 0.737 | -0.03 | 0.905 |
| cCSA | 0.05 | 0.835 | -0.06 | 0.793 | -0.15 | 0.523 | -0.23 | 0.313 | 0.40 | 0.093 | 0.36 | 0.099 | 0.41 | 0.072 | 0.29 | 0.187 |
| ∆FF | 0.22 | 0.346 | 0.10 | 0.651 | 0.01 | 0.975 | 0.37 | 0.090 | 0.26 | 0.268 | 0.71 | 0.755 | 0.13 | 0.582 | 0.26 | 0.253 |
| ∆cCSA_rel_ | 0.10 | 0.673 | 0.13 | 0.577 | -0.12 | 0.618 | -0.18 | 0.425 | 0.06 | 0.801 | -0.18 | 0.425 | -0.14 | 0.561 | -0.16 | 0.465 |
| **TR** |  |  |  |  |  |  |  |  |  |  |  |  |  |  |  |  |
| FF | 0.06 | 0.801 | -0.17 | 0.453 | 0.47 | 0.036 | 0.10 | 0.651 | 0.42 | 0.062 | 0.47 | 0.029 | 0.03 | 0.915 | 0.21 | 0.353 |
| water T_2_ | 0.16 | 0.523 | 0.16 | 0.514 | 0.41 | 0.084 | -0.04 | 0.864 | 0.31 | 0.190 | 0.22 | 0.367 | -0.02 | 0.932 | -0.006 | 0.978 |
| cCSA | -0.09 | 0.696 | -0.02 | 0.934 | -0.42 | 0.062 | -0.09 | 0.687 | -0.002 | 0.995 | 0.10 | 0.662 | 0.44 | 0.054 | 0.32 | 0.145 |
| ∆FF | 0.30 | 0.198 | -0.27 | 0.217 | 0.34 | 0.137 | 0.12 | 0.597 | 0.31 | 0.184 | -0.22 | 0.323 | -0.02 | 0.940 | 0.27 | 0.220 |
| ∆cCSA_rel_ | -0.39 | 0.091 | 0.27 | 0.229 | -0.03 | 0.915 | -0.21 | 0.357 | -0.11 | 0.640 | -0.27 | 0.232 | -0.22 | 0.342 | -0.16 | 0.488 |
| **LE** |  |  |  |  |  |  |  |  |  |  |  |  |  |  |  |  |
| FF | 0.17 | 0.461 | -0.06 | 0.782 | 0.51 | 0.019 | 0.06 | 0.786 | 0.47 | 0.032 | 0.31 | 0.159 | -0.19 | 0.417 | 0.09 | 0.676 |
| water T_2_ | 0.23 | 0.336 | 0.19 | 0.420 | 0.41 | 0.073 | 0.12 | 0.613 | **0.55** | **0.012** | 0.26 | 0.277 | -0.008 | 0.975 | -0.03 | 0.885 |
| cCSA | -0.07 | 0.771 | -0.05 | 0.820 | -0.45 | 0.041 | -0.09 | 0.676 | -0.04 | 0.858 | 0.25 | 0.259 | 0.46 | 0.035 | 0.32 | 0.144 |
| ∆FF | 0.17 | 0.471 | -0.07 | 0.774 | 0.38 | 0.089 | 0.24 | 0.291 | 0.06 | 0.793 | -0.22 | 0.321 | 0.24 | 0.300 | 0.29 | 0.186 |
| ∆cCSA_rel_ | -0.16 | 0.482 | 0.31 | 0.159 | -0.16 | 0.496 | -0.29 | 0.196 | 0.10 | 0.662 | -0.23 | 0.301 | -0.18 | 0.434 | -0.13 | 0.561 |
| **QU** |  |  |  |  |  |  |  |  |  |  |  |  |  |  |  |  |
| FF | -0.19 | 0.417 | -0.05 | 0.828 | **0.67** | **0.001** | 0.06 | 0.782 | -0.05 | 0.845 | 0.13 | 0.580 | -0.33 | 0.139 | -0.27 | 0.225 |
| water T_2_ | 0.10 | 0.683 | -0.19 | 0.401 | 0.17 | 0.478 | -0.37 | 0.099 | 0.07 | 0.754 | 0.31 | 0.179 | 0.20 | 0.397 | 0.30 | 0.186 |
| cCSA | 0.18 | 0.443 | -0.14 | 0.549 | -0.50 | 0.024 | 0.02 | 0.934 | 0.35 | 0.126 | 0.49 | 0.021 | 0.38 | 0.099 | 0.09 | 0.704 |
| ∆FF | 0.37 | 0.095 | -0.41 | 0.059 | 0.17 | 0.462 | 0.06 | 0.778 | 0.40 | 0.069 | -0.03 | 0.879 | 0.35 | 0.118 | 0.37 | 0.087 |
| ∆cCSA_rel_ | -0.19 | 0.416 | **0.53** | **0.011** | -0.08 | 0.753 | 0.10 | 0.673 | -0.21 | 0.376 | -0.30 | 0.172 | **-0.58** | **0.008** | -0.30 | 0.172 |
| **HS** |  |  |  |  |  |  |  |  |  |  |  |  |  |  |  |  |
| FF | 0.03 | 0.902 | -0.11 | 0.629 | **0.57** | **0.007** | 0.13 | 0.559 | 0.21 | 0.351 | 0.40 | 0.064 | -0.47 | 0.032 | 0.21 | 0.353 |
| water T_2_ | 0.27 | 0.232 | 0.15 | 0.526 | 0.36 | 0.111 | 0.23 | 0.328 | 0.12 | 0.602 | 0.44 | 0.046 | -0.08 | 0.741 | -0.006 | 0.978 |
| cCSA | -0.04 | 0.875 | -0.12 | 0.594 | -0.49 | 0.030 | 0.08 | 0.721 | 0.16 | 0.502 | 0.42 | 0.052 | 0.52 | 0.018 | 0.32 | 0.145 |
| ∆FF | 0.08 | 0.720 | -0.07 | 0.747 | 0.19 | 0.401 | 0.38 | 0.081 | 0.06 | 0.806 | -0.05 | 0.832 | -0.19 | 0.401 | 0.27 | 0.220 |
| ∆cCSA_rel_ | -0.34 | 0.146 | 0.16 | 0.490 | -0.15 | 0.531 | 0.04 | 0.871 | -0.12 | 0.605 | -0.43 | 0.017 | 0.05 | 0.840 | -0.16 | 0.488 |
| **TH** |  |  |  |  |  |  |  |  |  |  |  |  |  |  |  |  |
| FF | -0.23 | 0.326 | -0.06 | 0.814 | **0.67** | **0.001** | 0.01 | 0.960 | 0.05 | 0.850 | 0.30 | 0.182 | -0.37 | 0.110 | 0.29 | 0.189 |
| water T_2_ | 0.23 | 0.336 | -0.08 | 0.73 | 0.17 | 0.478 | 0.10 | 0.691 | 0.25 | 0.280 | 0.41 | 0.075 | 0.18 | 0.450 | 0.12 | 0.613 |
| cCSA | 0.22 | 0.352 | -0.13 | 0.586 | -0.50 | 0.024 | 0.01 | 0.973 | 0.36 | 0.118 | **0.60** | **0.004** | 0.45 | 0.047 | 0.13 | 0.571 |
| ∆FF | 0.33 | 0.150 | -0.33 | 0.143 | 0.17 | 0.462 | 0.01 | 0.951 | 0.11 | 0.654 | -0.12 | 0.598 | 0.15 | 0.523 | 0.12 | 0.606 |
| ∆cCSA_rel_ | -0.47 | 0.038 | 0.40 | 0.072 | -0.08 | 0.753 | 0.24 | 0.302 | -0.12 | 0.613 | -0.39 | 0.078 | -0.27 | 0.251 | -0.28 | 0.224 |

Significant differences are indicated in bold (moderate correlations in **light grey**, strong correlations in **dark grey**). BL, baseline; BMI, body-mass index (kg/m^2^); cCSA, contractile cross-sectional area (cm^2^); CK, creatine kinease contration (U/L); EX, *extensor*; FF, fat fraction (%); HS, hamstring; LE, global leg; QU, *quadriceps*; TH, global thigh; TR, *triceps surae*; ρ, Spearman-rank correlation coefficient; ∆cCSA_rel_, one-year relative change in cCSA (%); ∆FF, one-year change in FF (%); 6MWD, six-minute walking distance (in m).

**B. Functional/strength parameters: 6MWD**

|  | **Correlation with**  **6MWD at BL**  **placebo**  **(m)** | | **Correlation with**  **6MWD at BL**  **placebo**  **(%pred)** | | **Correlation with**  **6MWD at BL**  **sirolimus**  **(m)** | | **Correlation with**  **6MWD at BL**  **sirolimus**  **(%pred)** | | **Correlation with**  **∆6MWD**  **placebo**  **(m)** | | **Correlation with**  **∆6MWD**  **placebo**  **(%pred)** | | **Correlation with**  **∆6MWD**  **sirolimus**  **(m)** | | **Correlation with**  **∆6MWD**  **sirolimus**  **(%pred)** | |
| --- | --- | --- | --- | --- | --- | --- | --- | --- | --- | --- | --- | --- | --- | --- | --- | --- |
|  | *ρ* | *P* | *ρ* | *P* | *ρ* | *P* | *ρ* | *P* | *ρ* | *P* | *ρ* | *P* | *ρ* | *P* | *ρ* | *P* |
| **EX** |  |  |  |  |  |  |  |  |  |  |  |  |  |  |  |  |
| FF | **-0.54** | **0.015** | **-0.55** | **0.013** | **-0.69** | **<0.001** | **-0.57** | **0.006** | -0.37 | 0.135 | -0.45 | 0.063 | 0.02 | 0.935 | -0.03 | 0.911 |
| water T_2_ | -0.45 | 0.056 | -0.40 | 0.093 | -0.27 | 0.287 | -0.43 | 0.058 | -0.31 | 0.220 | -0.35 | 0.168 | -0.04 | 0.881 | -0.02 | 0.926 |
| cCSA | 0.34 | 0.145 | 0.34 | 0.141 | 0.46 | 0.038 | 0.19 | 0.396 | 0.12 | 0.645 | 0.16 | 0.537 | -0.11 | 0.663 | -0.02 | 0.942 |
| ∆FF | -0.04 | 0.875 | -0.07 | 0.777 | -0.32 | 0.171 | -0.39 | 0.075 | **-0.60** | **0.009** | **-0.57** | **0.013** | -0.17 | 0.488 | -0.12 | 0.610 |
| ∆cCSA_rel_ | 0.01 | 0.980 | 0.11 | 0.650 | -0.04 | 0.855 | 0.28 | 0.208 | 0.16 | 0.521 | 0.21 | 0.399 | 0.40 | 0.088 | 0.22 | 0.330 |
| **TR** |  |  |  |  |  |  |  |  |  |  |  |  |  |  |  |  |
| FF | **-0.62** | **0.004** | **-0.61** | **0.004** | **-0.72** | **<0.001** | **-0.75** | **<0.001** | -0.43 | 0.075 | -0.52 | 0.026 | 0.04 | 0.867 | -0.02 | 0.920 |
| water T_2_ | **-0.75** | **<0.001** | **-0.63** | **0.004** | -0.17 | 0.523 | -0.44 | 0.057 | -0.11 | 0.667 | -0.19 | 0.474 | 0.05 | 0.858 | 0.08 | 0.742 |
| cCSA | 0.46 | 0.041 | 0.33 | 0.152 | 0.48 | 0.032 | 0.31 | 0.167 | 0.02 | 0.938 | 0.09 | 0.729 | -0.14 | 0.579 | -0.13 | 0.563 |
| ∆FF | -0.46 | 0.040 | -0.42 | 0.066 | -0.10 | 0.970 | -0.05 | 0.844 | -0.48 | 0.050 | **-0.56** | **0.016** | -0.14 | 0.574 | -0.22 | 0.330 |
| ∆cCSA_rel_ | 0.16 | 0.506 | 0.12 | 0.622 | 0.14 | 0.571 | 0.22 | 0.342 | 0.17 | 0.505 | 0.26 | 0.299 | -0.02 | 0.926 | 0.05 | 0.836 |
| **LE** |  |  |  |  |  |  |  |  |  |  |  |  |  |  |  |  |
| FF | **-0.68** | **<0.001** | **-0.68** | **<0.001** | **-0.77** | **<0.001** | **-0.75** | **<0.001** | -0.36 | 0.128 | **-0.54** | **0.017** | 0.05 | 0.579 | 0.02 | 0.920 |
| water T_2_ | **-0.77** | **<0.001** | **-0.68** | **<0.001** | -0.36 | 0.140 | -0.52 | 0.019 | -0.07 | 0.785 | -0.26 | 0.303 | 0.07 | 0.833 | 0.05 | 0.847 |
| cCSA | **0.53** | **0.013** | 0.45 | 0.042 | 0.51 | 0.022 | 0.28 | 0.214 | 0.10 | 0.679 | 0.23 | 0.351 | -0.14 | 0.579 | -0.13 | 0.586 |
| ∆FF | -0.27 | 0.236 | -0.23 | 0.325 | -0.23 | 0.332 | -0.15 | 0.506 | **-0.60** | **0.008** | **-0.61** | **0.005** | -0.11 | 0.644 | -0.21 | 0.369 |
| ∆cCSA_rel_ | -0.01 | 0.969 | 0.01 | 0.973 | 0.03 | 0.910 | 0.29 | 0.195 | 0.20 | 0.424 | 0.20 | 0.412 | 0.13 | 0.611 | 0.03 | 0.907 |
| **QU** |  |  |  |  |  |  |  |  |  |  |  |  |  |  |  |  |
| FF | -0.50 | 0.021 | **-0.51** | **0.017** | -0.48 | 0.034 | -0.50 | 0.018 | -0.26 | 0.276 | -0.34 | 0.149 | -0.22 | 0.371 | -0.30 | 0.188 |
| water T_2_ | -0.44 | 0.046 | -0.42 | 0.058 | 0.23 | 0.336 | -0.13 | 0.567 | -0.29 | 0.223 | -0.38 | 0.106 | -0.29 | 0.245 | -0.14 | 0.552 |
| cCSA | 0.31 | 0.182 | 0.27 | 0.251 | 0.26 | 0.262 | 0.01 | 0.958 | 0.04 | 0.874 | 0.04 | 0.874 | 0.22 | 0.365 | 0.40 | 0.073 |
| ∆FF | -0.32 | 0.162 | -0.24 | 0.291 | 0.12 | 0.609 | 0.12 | 0.604 | **-0.62** | **0.005** | **-0.70** | **<0.001** | -0.23 | 0.340 | -0.19 | 0.410 |
| ∆cCSA_rel_ | -0.04 | 0.865 | 0.003 | 0.990 | -0.30 | 0.205 | -0.08 | 0.732 | 0.22 | 0.390 | 0.31 | 0.210 | -0.04 | 0.861 | -0.21 | 0.354 |
| **HS** |  |  |  |  |  |  |  |  |  |  |  |  |  |  |  |  |
| FF | **-0.74** | **<0.001** | **-0.71** | **<0.001** | **-0.58** | **0.008** | **-0.72** | **<0.001** | -0.07 | 0.792 | -0.19 | 0.429 | -0.004 | 0.989 | 0.04 | 0.854 |
| water T_2_ | **-0.58** | **0.006** | -0.48 | 0.027 | 0.16 | 0.509 | -0.08 | 0.724 | 0.17 | 0.495 | 0.002 | 0.994 | -0.20 | 0.425 | -0.07 | 0.772 |
| cCSA | **0.53** | **0.016** | 0.46 | 0.043 | 0.18 | 0.450 | 0.15 | 0.506 | -0.24 | 0.332 | -0.17 | 0.499 | 0.15 | 0.540 | -0.08 | 0.716 |
| ∆FF | -0.36 | 0.115 | -0.34 | 0.148 | 0.40 | 0.084 | 0.15 | 0.516 | -0.17 | 0.495 | -0.20 | 0.412 | -0.43 | 0.064 | -0.30 | 0.192 |
| ∆cCSA_rel_ | 0.35 | 0.126 | 0.25 | 0.292 | -0.34 | 0.141 | -0.15 | 0.500 | -0.17 | 0.505 | 0.04 | 0.868 | 0.20 | 0.407 | 0.03 | 0.893 |
| **TH** |  |  |  |  |  |  |  |  |  |  |  |  |  |  |  |  |
| FF | **-0.65** | **0.002** | **-0.68** | **0.001** | **-0.71** | **<0.001** | **-0.81** | **<0.001** | -0.10 | 0.687 | -0.25 | 0.320 | -0.001 | 0.997 | -0.06 | 0.811 |
| water T_2_ | -0.47 | 0.039 | -0.41 | 0.071 | 0.46 | 0.053 | 0.04 | 0.855 | 0.10 | 0.693 | -0.97 | 0.779 | -0.20 | 0.442 | -0.03 | 0.904 |
| cCSA | 0.36 | 0.121 | 0.32 | 0.164 | 0.39 | 0.096 | 0.22 | 0.336 | -0.12 | 0.645 | -0.08 | 0.760 | 0.08 | 0.741 | 0.14 | 0.548 |
| ∆FF | -0.39 | 0.092 | -0.13 | 0.436 | 0.22 | 0.363 | 0.27 | 0.234 | -0.55 | <0.018 | **-0.66** | **0.003** | -0.46 | 0.053 | -0.38 | 0.101 |
| ∆cCSA_rel_ | 0.33 | 0.150 | 0.15 | 0.361 | -0.39 | 0.098 | -0.23 | 0.328 | 0.27 | 0.280 | 0.47 | 0.050 | 0.07 | 0.779 | -0.15 | 0.527 |

Significant differences are indicated in bold (moderate correlations in **light grey**, strong correlations in **dark grey**). BL, baseline; cCSA, contractile cross-sectional area (cm^2^); EX, *extensor*; FF, fat fraction (%); HS, hamstring; LE, global leg; QU, *quadriceps*; TH, global thigh; TR, *triceps surae*; ρ, Spearman-rank correlation coefficient; ∆cCSA_rel_, one-year relative change in cCSA (%); ∆FF, one-year change in FF (%); 6MWD, six-minute walking distance (in m and %pred); ∆6MWD, one-year change in six-minute walking distance (in m and %pred).

**C. Functional/strength parameters: Knee extension**

|  | **Correlation with**  **knee extension strength at BL**  **(Nm)**  **placebo** | | **Correlation with**  **knee extension strength at BL (%pred)**  **placebo** | | **Correlation with**  **knee extension strength at BL**  **(Nm)**  **sirolimus** | | **Correlation with**  **knee extension strength at BL (%pred)**  **sirolimus** | | **Correlation with**  **∆knee extension strength (Nm)**  **placebo** | | **Correlation with**  **∆knee extension strength**  **(%pred)**  **placebo** | | **Correlation with**  **∆knee extension strength (Nm)**  **sirolimus** | | **Correlation with**  **∆knee extension strength**  **(%pred) sirolimus** | |
| --- | --- | --- | --- | --- | --- | --- | --- | --- | --- | --- | --- | --- | --- | --- | --- | --- |
|  | *ρ* | *P* | *ρ* | *P* | *ρ* | *P* | *ρ* | *P* | *ρ* | *P* | *ρ* | *P* | *ρ* | *P* | *ρ* | *P* |
| **EX** |  |  |  |  |  |  |  |  |  |  |  |  |  |  |  |  |
| FF | -0.21 | 0.376 | -0.19 | 0.413 | -0.23 | 0.311 | -0.24 | 0.287 | -0.03 | 0.192 | -0.02 | 0.950 | 0.30 | 0.192 | 0.32 | 0.158 |
| water T_2_ | -0.20 | 0.408 | -0.18 | 0.455 | -0.10 | 0.677 | -0.10 | 0.686 | -0.05 | 0.847 | -0.05 | 0.825 | 0.10 | 0.700 | 0.12 | 0.616 |
| cCSA | 0.32 | 0.169 | 0.32 | 0.139 | 0.09 | 0.687 | 0.11 | 0.633 | -0.39 | 0.086 | -0.37 | 0.112 | -0.26 | 0.265 | -0.29 | 0.205 |
| ∆FF | 0.11 | 0.654 | 0.16 | 0.502 | **-0.56** | **0.007** | **-0.52** | **0.013** | -0.22 | 0.342 | -0.17 | 0.470 | 0.24 | 0.291 | 0.15 | 0.529 |
| ∆cCSA_rel_ | 0.12 | 0.609 | 0.07 | 0.758 | 0.42 | 0.051 | 0.40 | 0.063 | 0.02 | 0.940 | -0.05 | 0.830 | -0.14 | 0.559 | -0.14 | 0.548 |
| **TR** |  |  |  |  |  |  |  |  |  |  |  |  |  |  |  |  |
| FF | **-0.62** | **0.004** | **-0.57** | **0.009** | -0.30 | 0.186 | -0.33 | 0.136 | 0.09 | 0.719 | 0.12 | 0.605 | 0.30 | 0.192 | 0.39 | 0.080 |
| water T_2_ | **-0.57** | **0.011** | **-0.58** | **0.009** | -0.39 | 0.099 | -0.39 | 0.101 | 0.24 | 0.314 | 0.24 | 0.329 | 0.15 | 0.542 | 0.13 | 0.616 |
| cCSA | **0.70** | **<0.001** | **0.71** | **<0.001** | 0.13 | 0.566 | 0.16 | 0.477 | -0.39 | 0.091 | -0.36 | 0.115 | -0.07 | 0.763 | -0.20 | 0.394 |
| ∆FF | -0.33 | 0.162 | -0.29 | 0.214 | -0.49 | 0.019 | -0.45 | 0.037 | -0.06 | 0.816 | 0.01 | 0.975 | 0.25 | 0.273 | 0.16 | 0.500 |
| ∆cCSA_rel_ | 0.15 | 0.539 | 0.12 | 0.622 | 0.04 | 0.854 | -0.004 | 0.987 | 0.24 | 0.310 | 0.20 | 0.398 | -0.07 | 0.780 | -0.11 | 0.634 |
| **LE** |  |  |  |  |  |  |  |  |  |  |  |  |  |  |  |  |
| FF | -0.43 | 0.052 | -0.38 | 0.092 | -0.36 | 0.098 | -0.39 | 0.074 | 0.19 | 0.404 | 0.28 | 0.211 | 0.25 | 0.278 | 0.32 | 0.153 |
| water T_2_ | -0.41 | 0.071 | -0.43 | 0.060 | -0.26 | 0.271 | -0.27 | 0.257 | 0.27 | 0.257 | 0.32 | 0.173 | 0.22 | 0.375 | 0.26 | 0.290 |
| cCSA | **0.65** | **0.002** | **0.63** | **0.002** | 0.16 | 0.490 | 0.19 | 0.393 | -0.45 | 0.041 | -0.48 | 0.028 | -0.10 | 0.670 | -0.20 | 0.391 |
| ∆FF | -0.48 | 0.029 | -0.47 | 0.033 | -0.36 | 0.096 | -0.37 | 0.086 | -0.11 | 0.642 | -0.09 | 0.695 | 0.32 | 0.153 | 0.21 | 0.366 |
| ∆cCSA_rel_ | 0.10 | 0.683 | 0.07 | 0.750 | 0.31 | 0.162 | 0.32 | 0.152 | 0.39 | 0.084 | 0.34 | 0.138 | -0.07 | 0.767 | -0.13 | 0.563 |
| **QU** |  |  |  |  |  |  |  |  |  |  |  |  |  |  |  |  |
| FF | **-0.77** | **<0.001** | **-0.74** | **<0.001** | **-0.64** | **0.001** | **-0.64** | **0.001** | **0.55** | **0.010** | **0.58** | **0.006** | 0.19 | 0.417 | 0.11 | 0.650 |
| water T_2_ | **-0.62** | **0.003** | **-0.59** | **0.005** | -0.21 | 0.357 | -0.18 | 0.427 | 0.28 | 0.211 | 0.28 | 0.225 | -0.22 | 0.356 | -0.16 | 0.490 |
| cCSA | **0.87** | **<0.001** | **0.88** | **<0.001** | **0.62** | **0.002** | **0.58** | **0.004** | **-0.58** | **0.007** | **-0.54** | **0.014** | -0.23 | 0.325 | -0.14 | 0.537 |
| ∆FF | -0.15 | 0.518 | -0.11 | 0.642 | -0.13 | 0.577 | -0.13 | 0.570 | -0.21 | 0.354 | -0.18 | 0.427 | -0.19 | 0.420 | -0.30 | 0.192 |
| ∆cCSA_rel_ | -0.13 | 0.578 | -0.18 | 0.443 | -0.28 | 0.216 | -0.24 | 0.291 | 0.50 | 0.026 | 0.47 | 0.038 | **0.54** | **0.011** | 0.47 | 0.033 |
| **HS** |  |  |  |  |  |  |  |  |  |  |  |  |  |  |  |  |
| FF | **-0.59** | **0.005** | **-0.57** | **0.007** | -0.35 | 0.106 | -0.41 | 0.056 | 0.50 | 0.023 | **0.53** | **0.014** | 0.08 | 0.746 | 0.05 | 0.841 |
| water T_2_ | -0.43 | 0.052 | -0.45 | 0.043 | 0.15 | 0.529 | 0.14 | 0.552 | 0.46 | 0.038 | 0.46 | 0.035 | -0.03 | 0.910 | -0.13 | 0.582 |
| cCSA | **0.53** | **0.017** | **0.54** | **0.013** | 0.34 | 0.118 | 0.35 | 0.111 | -0.49 | 0.028 | -0.49 | 0.029 | 0.21 | 0.360 | 0.21 | 0.360 |
| ∆FF | -0.40 | 0.074 | -0.39 | 0.089 | -0.20 | 0.363 | -0.19 | 0.402 | 0.16 | 0.478 | 0.205 | 0.372 | -0.02 | 0.938 | -0.18 | 0.430 |
| ∆cCSA_rel_ | 0.23 | 0.323 | 0.22 | 0.349 | -0.23 | 0.299 | -0.23 | 0.301 | -0.05 | 0.830 | -0.16 | 0.498 | 0.29 | 0.201 | 0.22 | 0.336 |
| **TH** |  |  |  |  |  |  |  |  |  |  |  |  |  |  |  |  |
| FF | **-0.74** | **<0.001** | **-0.71** | **<0.001** | **-0.60** | **0.004** | **-0.64** | **0.002** | 0.45 | 0.045 | 0.52 | 0.018 | 0.23 | 0.336 | 0.21 | 0.376 |
| water T_2_ | -0.25 | 0.286 | -0.25 | 0.292 | 0.25 | 0.283 | 0.23 | 0.332 | 0.19 | 0.424 | 0.24 | 0.307 | -0.11 | 0.663 | -0.11 | 0.632 |
| cCSA | **0.89** | **<0.001** | **0.88** | **<0.001** | **0.60** | **0.004** | **0.58** | **0.006** | **-0.53** | **0.016** | -0.51 | 0.023 | -0.23 | 0.329 | -0.20 | 0.409 |
| ∆FF | -0.32 | 0.169 | -0.25 | 0.286 | -0.07 | 0.758 | -0.03 | 0.884 | -0.23 | 0.329 | -0.17 | 0.466 | -0.16 | 0.510 | -0.27 | 0.248 |
| ∆cCSA_rel_ | 0.24 | 0.301 | 0.19 | 0.427 | 0.32 | 0.156 | -0.33 | 0.141 | 0.17 | 0.478 | 0.10 | 0.686 | 0.49 | 0.028 | 0.42 | 0.064 |

Significant differences are indicated in bold (moderate correlations in **light grey**, strong correlations in **dark grey**, very strong correlations in **black**). BL, baseline; cCSA, contractile cross-sectional area (cm^2^); EX, *extensor*; FF, fat fraction (%); HS, hamstring; LE, global leg; QU, *quadriceps*; TH, global thigh; TR, *triceps surae*; ρ, Spearman-rank correlation coefficient; ∆cCSA_rel_, one-year relative change in cCSA (%); ∆FF, one-year change in FF (%).

**D. Functional/strength parameters: Knee flexion**

|  | **Correlation with**  **knee flexion strength at BL**  **(Nm)**  **placebo** | | **Correlation with**  **knee flexion strength at BL (%pred)**  **placebo** | | **Correlation with**  **knee flexion strength at BL**  **(Nm)**  **sirolimus** | | **Correlation with**  **knee flexion strength at BL (%pred)**  **sirolimus** | | **Correlation with**  **∆knee flexion strength**  **(Nm)**  **placebo** | | **Correlation with**  **∆knee flexion strength (%pred)**  **placebo** | | | **Correlation with**  **∆knee flexion strength**  **(Nm)**  **sirolimus** | | | **Correlation with**  **∆knee flexion strength (%pred)**  **sirolimus** | | |
| --- | --- | --- | --- | --- | --- | --- | --- | --- | --- | --- | --- | --- | --- | --- | --- | --- | --- | --- | --- |
|  | *ρ* | *P* | *ρ* | *P* | *ρ* | *P* | *ρ* | *P* | *ρ* | *P* | *ρ* | *P* | *ρ* | | *P* | *ρ* | | *P* |  |
| **EX** |  |  |  |  |  |  |  |  |  |  |  |  |  | |  |  | |  |  |
| FF | -0.42 | 0.057 | -0.34 | 0.143 | -0.47 | 0.028 | -0.50 | 0.018 | 0.26 | 0.268 | 0.24 | 0.307 | 0.11 | | 0.642 | 0.06 | | 0.788 |  |
| water T_2_ | -0.45 | 0.055 | -0.40 | 0.090 | -0.49 | 0.027 | -0.44 | 0.053 | 0.36 | 0.132 | 0.29 | 0.232 | -0.02 | | 0.932 | -0.10 | | 0.700 |  |
| cCSA | 0.32 | 0.175 | 0.26 | 0.265 | 0.27 | 0.221 | 0.32 | 0.146 | -0.45 | 0.049 | -0.37 | 0.107 | -0.04 | | 0.849 | -0.04 | | 0.862 |  |
| ∆FF | -0.13 | 0.600 | -0.02 | 0.950 | -0.44 | 0.042 | -0.44 | 0.040 | 0.07 | 0.782 | 0.01 | 0.970 | -0.20 | | 0.378 | -0.29 | | 0.207 |  |
| ∆cCSA_rel_ | -0.01 | 0.965 | -0.01 | 0.965 | 0.09 | 0.680 | 0.14 | 0.533 | 0.36 | 0.125 | 0.23 | 0.339 | 0.24 | | 0.294 | 0.32 | | 0.160 |  |
| **TR** |  |  |  |  |  |  |  |  |  |  |  |  |  | |  |  | |  |  |
| FF | **-0.56** | **0.010** | **-0.55** | **0.011** | **-0.67** | **<0.001** | **-0.75** | **<0.001** | 0.25 | 0.295 | 0.25 | 0.295 | 0.45 | | 0.042 | 0.39 | | 0.081 |  |
| water T_2_ | **-0.79** | **<0.001** | **-0.79** | **<0.001** | -0.51 | 0.026 | -0.48 | 0.037 | 0.29 | 0.235 | 0.25 | 0.293 | -0.18 | | 0.478 | -0.25 | | 0.324 |  |
| cCSA | 0.44 | 0.055 | 0.50 | 0.024 | **0.59** | **0.004** | **0.63** | **0.002** | -0.27 | 0.248 | -0.25 | 0.289 | -0.23 | | 0.311 | -0.22 | | 0.336 |  |
| ∆FF | -0.49 | 0.029 | -0.41 | 0.073 | -0.26 | 0.248 | -0.20 | 0.366 | -0.05 | 0.840 | -0.07 | 0.772 | 0.07 | | 0.754 | -0.02 | | 0.938 |  |
| ∆cCSA_rel_ | 0.02 | 0.945 | 0.03 | 0.915 | 0.20 | 0.391 | 0.18 | 0.427 | 0.50 | 0.025 | 0.42 | 0.062 | 0.07 | | 0.750 | 0.09 | | 0.703 |  |
| **LE** |  |  |  |  |  |  |  |  |  |  |  |  |  | |  |  | |  |  |
| FF | **-0.59** | **0.005** | **-0.54** | **0.011** | **-0.75** | **<0.001** | **-0.81** | **<0.001** | 0.38 | 0.094 | 0.37 | 0.095 | 0.35 | | 0.116 | 0.30 | | 0.192 |  |
| water T_2_ | **-0.77** | **<0.001** | **-0.73** | **<0.001** | **-0.54** | **0.015** | **-0.55** | **0.013** | 0.41 | 0.076 | 0.39 | 0.086 | -0.08 | | 0.759 | -0.13 | | 0.606 |  |
| cCSA | 0.51 | 0.018 | **0.56** | **0.009** | **0.55** | **0.009** | **0.60** | **0.004** | -0.33 | 0.146 | -0.33 | 0.151 | -0.19 | | 0.414 | -0.16 | | 0.478 |  |
| ∆FF | -0.24 | 0.294 | -0.26 | 0.256 | -0.42 | 0.052 | -0.32 | 0.142 | -0.37 | 0.103 | -0.35 | 0.125 | 0.14 | | 0.559 | 0.04 | | 0.858 |  |
| ∆cCSA_rel_ | -0.10 | 0.683 | -0.06 | 0.788 | 0.39 | 0.073 | 0.40 | 0.068 | **0.71** | **<0.001** | **0.64** | **0.002** | 0.16 | | 0.496 | 0.24 | | 0.300 |  |
| **QU** |  |  |  |  |  |  |  |  |  |  |  |  |  | |  |  | |  |  |
| FF | **-0.54** | **0.011** | **-0.54** | **0.011** | **-0.54** | **0.011** | -0.37 | 0.095 | 0.35 | 0.122 | 0.36 | 0.113 | 0.03 | | 0.915 | -0.02 | | 0.933 |  |
| water T_2_ | -0.48 | 0.026 | -0.44 | 0.047 | -0.48 | 0.026 | 0.14 | 0.540 | 0.07 | 0.754 | 0.04 | 0.849 | -0.21 | | 0.380 | -0.21 | | 0.366 |  |
| cCSA | 0.39 | 0.092 | 0.49 | 0.029 | 0.39 | 0.092 | 0.24 | 0.273 | -0.17 | 0.462 | -0.19 | 0.439 | -0.16 | | 0.482 | -0.13 | | 0.563 |  |
| ∆FF | -0.25 | 0.278 | -0.19 | 0.401 | -0.25 | 0.278 | 0.16 | 0.474 | -0.12 | 0.598 | -0.15 | 0.507 | -0.19 | | 0.414 | -0.21 | | 0.363 |  |
| ∆cCSA_rel_ | -0.24 | 0.301 | -0.23 | 0.332 | -0.24 | 0.301 | 0.13 | 0.563 | 0.52 | 0.020 | 0.42 | 0.069 | 0.13 | | 0.563 | 0.09 | | 0.691 |  |
| **HS** |  |  |  |  |  |  |  |  |  |  |  |  |  | |  |  | |  |  |
| FF | **-0.80** | **<0.001** | **-0.78** | **<0.001** | **-0.80** | **<0.001** | **-0.74** | **<0.001** | 0.47 | 0.030 | 0.51 | 0.018 | 0.19 | | 0.410 | 0.12 | | 0.602 |  |
| water T_2_ | **-0.66** | **0.001** | **-0.58** | **0.006** | **-0.66** | **0.001** | -0.01 | 0.955 | 0.29 | 0.203 | 0.26 | 0.256 | -0.08 | | 0.734 | -0.15 | | 0.527 |  |
| cCSA | **0.71** | **<0.001** | **0.70** | **<0.001** | **0.71** | **<0.001** | **0.57** | **0.006** | -0.32 | 0.173 | -0.30 | 0.200 | 0.11 | | 0.638 | 0.14 | | 0.552 |  |
| ∆FF | **-0.62** | **0.003** | **-0.56** | **0.009** | **-0.62** | **0.003** | 0.01 | 0.970 | 0.12 | 0.618 | 0.04 | 0.854 | -0.22 | | 0.339 | -0.34 | | 0.136 |  |
| ∆cCSA_rel_ | 0.24 | 0.310 | 0.25 | 0.286 | 0.24 | 0.310 | -0.05 | 0.832 | 0.34 | 0.145 | 0.24 | 0.307 | -0.09 | | 0.691 | -0.05 | | 0.841 |  |
| **TH** |  |  |  |  |  |  |  |  |  |  |  |  |  | |  |  | |  |  |
| FF | **-0.69** | **<0.001** | **-0.69** | **<0.001** | **-0.61** | **0.004** | **-0.68** | **<0.001** | 0.26 | 0.265 | 0.32 | 0.152 | 0.13 | | 0.600 | 0.09 | | 0.701 |  |
| water T_2_ | -0.51 | 0.020 | -0.43 | 0.056 | 0.20 | 0.402 | 0.23 | 0.332 | 0.12 | 0.605 | 0.15 | 0.523 | -0.26 | | 0.276 | -0.29 | | 0.232 |  |
| cCSA | 0.48 | 0.034 | **0.56** | **0.010** | **0.55** | **0.010** | **0.56** | **0.009** | -0.09 | 0.705 | -0.14 | 0.556 | -0.12 | | 0.605 | -0.09 | | 0.715 |  |
| ∆FF | -0.22 | 0.363 | -0.21 | 0.384 | 0.21 | 0.369 | 0.25 | 0.271 | -0.48 | 0.034 | -0.42 | 0.069 | -0.08 | | 0.748 | -0.14 | | 0.561 |  |
| ∆cCSA_rel_ | 0.15 | 0.539 | 0.14 | 0.569 | -0.28 | 0.223 | -0.28 | 0.223 | 0.45 | 0.043 | 0.39 | 0.094 | 0.09 | | 0.705 | 0.10 | | 0.673 |  |

Significant differences are indicated in bold (moderate correlations in **light grey**, strong correlations in **dark grey**, very strong correlations in **black**). BL, baseline; cCSA, contractile cross-sectional area (cm^2^); EX, *extensor*; FF, fat fraction (%); HS, hamstring; LE, global leg; QU, *quadriceps*; TH, global thigh; TR, *triceps surae*; ρ, Spearman-rank correlation coefficient; ∆cCSA_rel_, one-year relative change in cCSA (%); ∆FF, one-year change in FF (%).

**Supplementary Tables 11** Correlations ^31^P MRS-clinical/functional/strength parameters

**A. Clinical parameters**

|  | **Correlation with**  **age at BL** | | **Correlation with**  **#years since symptom onset at BL** | | **Correlation with**  **BMI at BL** | | **Correlation with**  **CK at BL** | |
| --- | --- | --- | --- | --- | --- | --- | --- | --- |
|  | *ρ* | *P* | *ρ* | *P* | *ρ* | *P* | *ρ* | *P* |
| **TRICEPS SURAE** |  |  |  |  |  |  |  |  |
| P_i,tot_/PCr | -0.17 | 0.430 | 0.36 | 0.089 | -0.05 | 0.837 | -0.18 | 0.433 |
| P_i,tot_/γATP | -0.32 | 0.140 | 0.23 | 0.297 | 0.25 | 0.246 | 0.18 | 0.412 |
| PCr/γATP | 0.48 | 0.020 | -0.12 | 0.590 | -0.40 | 0.056 | -0.43 | 0.039 |
| PDE/γATP | 0.06 | 0.792 | 0.31 | 0.154 | 0.31 | 0.148 | 0.17 | 0.444 |
| PME/γATP | -0.32 | 0.139 | 0.30 | 0.170 | 0.12 | 0.593 | 0.03 | 0.897 |
| P_i,b_/P_i,tot_ | -0.11 | 0.631 | -0.11 | 0.634 | 0.06 | 0.778 | 0.21 | 0.335 |
| pH_w_ | 0.15 | 0.500 | 0.21 | 0.342 | 0.33 | 0.128 | 0.32 | 0.139 |
| [Mg^2+^] (mM) | -0.03 | 0.904 | -0.24 | 0.266 | -0.17 | 0.449 | -0.50 | 0.014 |
| **QUADRICEPS** |  |  |  |  |  |  |  |  |
| P_i,tot_/PCr | 0.30 | 0.325 | 0.40 | 0.181 | 0.32 | 0.289 | 0.19 | 0.541 |
| P_i,tot_/γATP | 0.36 | 0.231 | 0.34 | 0.255 | 0.30 | 0.325 | 0.32 | 0.289 |
| PCr/γATP | -0.19 | 0.529 | -0.24 | 0.437 | -0.20 | 0.505 | -0.43 | 0.138 |
| PDE/γATP | 0.17 | 0.590 | 0.07 | 0.817 | 0.17 | 0.590 | -0.18 | 0.566 |
| PME/γATP | 0.13 | 0.681 | 0.35 | 0.247 | 0.04 | 0.887 | 0.50 | 0.086 |
| P_i,b_/P_i,tot_ | 0.10 | 0.734 | -0.08 | 0.803 | -0.35 | 0.247 | 0.47 | 0.108 |
| pH_w_ | 0.02 | 0.957 | -0.17 | 0.590 | -0.02 | 0.943 | 0.62 | 0.025 |
| [Mg^2+^] (mM) | 0.48 | 0.233 | 0.41 | 0.320 | 0.14 | 0.736 | -0.52 | 0.139 |

Significant differences are indicated in bold. BL, baseline; BMI, body-mass index (kg/m^2^); CK, creatine kinease contration (U/L); PCr, phosphocreatine; PDE, phosphodiesters; pH_w_, weighted pH; P_i,b_, alkaline inorganic phosphate; P_i,tot_, total inorganic phosphate; PME, phosphomonesters; γATP, adenosine triphosphate (γ-resonance in ^31^P MR spectrum); [Mg^2+^], intramuscular magnesium concentration (mM); ρ, Spearman-rank correlation coefficient; 6MWD, six-minute walking distance (in m).

**B. Functional/strength parameters**

|  | **Correlation with**  **6MWD at BL**  **(m)** | | **Correlation with**  **6MWD at BL**  **(%pred)** | | **Correlation with**  **∆6MWD**  **(m)** | | **Correlation with**  **∆6MWD**  **(%pred)** | | **Correlation with**  **knee extension strength at BL**  **(Nm)** | | **Correlation with**  **knee extension strength at BL**  **(%pred)** | | **Correlation with**  **∆knee extension strength**  **(Nm)** | | **Correlation with**  **∆knee extension strength**  **(%pred)** | |
| --- | --- | --- | --- | --- | --- | --- | --- | --- | --- | --- | --- | --- | --- | --- | --- | --- |
|  | *ρ* | *P* | *ρ* | *P* | *ρ* | *P* | *ρ* | *P* | *ρ* | *P* | *ρ* | *P* | *ρ* | *P* | *ρ* | *P* |
| **TRICEPS SURAE** |  |  |  |  |  |  |  |  |  |  |  |  |  |  |  |  |
| P_i,tot_/PCr | -0.34 | 0.136 | -0.16 | 0.474 | -0.39 | 0.097 | -0.23 | 0.313 | -0.49 | 0.018 | -0.40 | 0.061 | 0.37 | 0.091 | 0.35 | 0.115 |
| P_i,tot_/γATP | -0.28 | 0.225 | -0.20 | 0.356 | -0.11 | 0.668 | -0.49 | 0.025 | -0.37 | 0.087 | -0.50 | 0.015 | 0.25 | 0.259 | **0.58** | **0.004** |
| PCr/γATP | 0.21 | 0.366 | 0.10 | 0.660 | -0.11 | 0.664 | -0.01 | 0.951 | 0.17 | 0.446 | 0.21 | 0.326 | 0.04 | 0.867 | -0.09 | 0.699 |
| PDE/γATP | 0.13 | 0.580 | 0.03 | 0.907 | 0.02 | 0.929 | -0.10 | 0.654 | -0.06 | 0.781 | -0.04 | 0.865 | 0.41 | 0.056 | -0.13 | 0.573 |
| PME/γATP | **-0.58** | **0.005** | **-0.58** | **0.004** | -0.19 | 0.444 | -0.23 | 0.313 | **-0.64** | **0.001** | **-0.65** | **<0.001** | 0.54 | 0.010 | **0.66** | **<0.001** |
| P_i,b_/P_i,tot_ | 0.07 | 0.753 | -0.15 | 0.508 | 0.29 | 0.231 | 0.37 | 0.099 | 0.01 | 0.979 | 0.00 | 0.999 | -0.08 | 0.728 | -0.09 | 0.702 |
| pH_w_ | -0.02 | 0.940 | -0.26 | 0.229 | -0.16 | 0.510 | -0.02 | 0.942 | -0.05 | 0.823 | -0.02 | 0.936 | 0.06 | 0.789 | -0.10 | 0.658 |
| [Mg^2+^] (mM) | 0.27 | 0.231 | 0.43 | 0.042 | 0.18 | 0.454 | 0.10 | 0.658 | 0.36 | 0.09 | 0.38 | 0.071 | -0.09 | 0.680 | -0.32 | 0.154 |
| **QUADRICEPS** |  |  |  |  |  |  |  |  |  |  |  |  |  |  |  |  |
| P_i,tot_/PCr | -0.32 | 0.289 | -0.35 | 0.239 | -0.40 | 0.199 | -0.43 | 0.167 | -0.45 | 0.108 | -0.56 | 0.046 | 0.10 | 0.748 | 0.13 | 0.668 |
| P_i,tot_/γATP | -0.31 | 0.280 | -0.36 | 0.223 | -0.39 | 0.217 | -0.43 | 0.167 | -0.61 | 0.027 | -0.42 | 0.156 | 0.12 | 0.694 | 0.18 | 0.553 |
| PCr/γATP | 0.31 | 0.306 | 0.30 | 0.325 | 0.29 | 0.354 | -0.32 | 0.319 | 0.69 | 0.010 | **0.74** | **0.004** | -0.02 | 0.943 | 0.02 | 0.943 |
| PDE/γATP | 0.03 | 0.929 | -0.01 | 0.972 | -0.50 | 0.101 | -0.43 | 0.159 | -0.15 | 0.616 | -0.11 | 0.721 | -0.15 | 0.629 | -0.08 | 0.789 |
| PME/γATP | -0.21 | 0.494 | -0.26 | 0.394 | **-0.77** | **0.003** | -0.69 | 0.014 | -0.60 | 0.031 | -0.63 | 0.022 | -0.22 | 0.471 | -0.34 | 0.247 |
| P_i,b_/P_i,tot_ | -0.18 | 0.553 | -0.28 | 0.364 | -0.50 | 0.101 | -0.34 | 0.286 | -0.56 | 0.049 | -0.56 | 0.046 | -0.15 | 0.616 | -0.46 | 0.117 |
| pH_w_ | -0.01 | 0.986 | -0.12 | 0.707 | -0.49 | 0.106 | -0.41 | 0.183 | -0.29 | 0.334 | -0.29 | 0.344 | -0.15 | 0.616 | -0.39 | 0.194 |
| [Mg^2+^] (mM) | -0.29 | 0.493 | -0.12 | 0.779 | -0.17 | 0.693 | -0.24 | 0.570 | -0.10 | 0.823 | -0.05 | 0.911 | -0.02 | 0.955 | -0.17 | 0.693 |

Significant differences are indicated in bold (moderate correlations in **light grey**, strong correlations in **dark grey**)*.* BL, baseline; PCr, phosphocreatine; PDE, phosphodiesters; pH_w_, weighted pH; P_i,b_, alkaline inorganic phosphate; P_i,tot_, total inorganic phosphate; PME, phosphomonesters; γATP, adenosine triphosphate (γ-resonance in ^31^P MR spectrum); [Mg^2+^], intramuscular magnesium concentration (mM); ρ, Spearman-rank correlation coefficient; 6MWD, six-minute walking distance (in m).
